# UBL4A inhibits autophagy-mediated proliferation and metastasis of pancreatic ductal adenocarcinoma via targeting LAMP1

**DOI:** 10.1186/s13046-019-1278-9

**Published:** 2019-07-09

**Authors:** Hongze Chen, Le Li, Jisheng Hu, Zhongjie Zhao, Liang Ji, Chundong Cheng, Guangquan Zhang, Tao zhang, Yilong Li, Hua Chen, Shangha Pan, Bei Sun

**Affiliations:** 1 Department of Pancreatic and Biliary Surgery, The First Affiliated Hospital of Harbin Medical University, Harbin, 23 Youzheng Street, Nangang District, Harbin, 150001 Heilongjiang Province China; 2 Key Laboratory of Hepatosplenic Surgery, Ministry of Education, Harbin, Heilongjiang China; 3 Department of Breast Surgery, The First Affiliated Hospital of Harbin Medical University, Harbin, Heilongjiang China

**Keywords:** Ubiquitin-like protein 4A, UBL4A, Lysosome associated membrane protein-1, LAMP1, Autophagy, Lysosome dysfunction, Pancreatic ductal adenocarcinoma, PDAC, Proliferation and metastasis

## Abstract

**Background:**

Ubiquitin-like protein 4A (UBL4A) plays a significant role in protein metabolism and the maintenance of cellular homeostasis. In cancer, UBL4A represses tumorigenesis and is involved in various signaling pathways. Pancreatic ductal adenocarcinoma (PDAC) is still a major cause of cancer-related death and the underlying molecular mechanism of UBL4A and PDAC remains unknown.

**Methods:**

First, the prognostic role of UBL4A and its expression in human PDAC patients and in pancreatic cancer cell lines were detected by survival analysis and qRT-PCR, western blotting, and immunohistochemistry. Next, the effects of UBL4A on proliferation and metastasis in pancreatic cancer were evaluated by functional assays in vitro and in vivo. In addition, chloroquine was introduced to determine the role of autophagy in UBL4A-related tumor proliferation and metastasis. Ultimately, coimmunoprecipitation was used to confirm the interaction between UBL4A and lysosome associated membrane protein-1 (LAMP1), and western blotting was performed to explore the UBL4A mechanism.

**Results:**

We found that UBL4A was decreased in PDAC and that high levels of UBL4A correlated with a favorable prognosis. We observed that UBL4A inhibited tumor proliferation and metastasis through suppression of autophagy, a critical intracellular catabolic process that reportedly protects cells from nutrient starvation and other stress conditions. UBL4A caused impaired autophagic degradation in vitro, a crucial process in autophagy, by disturbing the function of lysosomes and contributing to autophagosome accumulation. We found a positive correlation between UBL4A and LAMP1. Furthermore, UBL4A caused lysosomal dysfunction by directly interacting with LAMP1, and LAMP1 overexpression reversed the antitumor effects of UBL4A in pancreatic cancer. In addition, we demonstrated that UBL4A suppressed tumor growth and metastasis in a pancreatic orthotopic tumor model.

**Conclusions:**

These findings suggest that UBL4A exerts an antitumor effect on autophagy-related proliferation and metastasis in PDAC by directly targeting LAMP1. Herein, we describe a novel mechanism of UBL4A that suppresses the progression of pancreatic cancer. UBL4A might be a promising target for the treatment and prognostication of PDAC.

**Electronic supplementary material:**

The online version of this article (10.1186/s13046-019-1278-9) contains supplementary material, which is available to authorized users.

## Background

Pancreatic ductal adenocarcinoma (PDAC) remains one of the most lethal malignant tumors. The 5-year overall survival rate of PDAC is only 9%, despite many large-scale efforts and continuous attempts to improve diagnosis and treatment [1]. The high mortality rate of pancreatic cancer patients is due to the difficulty in diagnosing the disease at an early stage, its tendency to metastasize and its drug resistance [2, 3]. Therefore, early diagnosis of PDAC and identification of appropriate therapeutic targets are of great importance.

Ubiquitin-like protein 4A (UBL4A) is a small protein composed of 157 amino acids located on the X chromosome (Xq28) [4]. Recent studies have indicated that UBL4A acts as a chaperone in protein processing in the endoplasmic reticulum [5, 6]. Other known functions of UBL4A include involvement in tumor suppression and in cell death in response to DNA damage [7], indicating the versatile capabilities of this protein. However, understanding of the exact biological function of UBL4A in the regulation of cancer cells, especially in PDAC, remains elusive [8].

Autophagy is a complex catabolic process that engulfs damaged proteins and organelles in autophagosomes and degrades them by fusion with lysosomes to protect cells from nutrient starvation and other stress conditions [9, 10]. Although autophagy was originally identified as a protective mechanism during starvation, it has also been associated with cell death [11]. Thus, autophagy is thought to underlie various processes in oncological diseases by modulating the initiation and/or maintenance of cancers [12, 13]. Lysosomes are responsible for the degradation of macromolecules derived from the extracellular space through endocytosis or phagocytosis, as well as from the cytoplasm through autophagy [14, 15]. Although autophagy has been well described as being directed by proteins encoded by autophagy-related genes (ATGs), there is increasing evidence that lysosomes are the central regulators of the autophagic process [16]. In addition, lysosomal membrane proteins may be involved in the interaction and fusion of lysosomes with autophagosomes, as well as in regulating the stability and integrity of the lysosome [17, 18].

Lysosome associated membrane protein-1 (LAMP1) and LAMP2 are major protein components of the lysosomal membrane. Both of these proteins were originally thought to protect the lysosomal membrane against the action of hydrolytic enzymes [19]. LAMP proteins are important regulators of the successful maturation of both autophagosomes and phagosomes. LAMP2 deficiency causes an accumulation of autophagosomes in many tissues, leading to cardiomyopathy and myopathy in mice and in patients suffering from Danon disease [20]. However, the role of LAMP1 in autophagy remains poorly understood.

In this study, we demonstrated that the elevated expression of UBL4A contributed to a favorable prognosis for PDAC patients and that UBL4A suppressed tumor growth and metastasis by inhibiting autophagy. In addition, we observed that UBL4A caused impaired autophagic degradation by disturbing the function of lysosomes through a direct interaction with LAMP1. Herein, we propose a novel mechanism by which UBL4A suppresses the autophagy-related proliferation and metastasis of PDAC.

## Methods

### Patients and specimens

PDAC and normal pancreatic tissues were obtained from 69 patients who underwent pancreatectomy in the Department of Pancreatic and Biliary Surgery (The First Affiliated Hospital of Harbin Medical University, Harbin, Heilongjiang, China) from January 2009 to January 2015. Informed consent was obtained from each patient prior to biopsy or surgery, and ethical approval for the use of human subjects was obtained from the Research Ethics Committee of the First Affiliated Hospital of Harbin Medical University. The patients’ clinical characteristics are shown in Table 1.

**Table 1:** Clinical correlation between UBL4A mRNA expression and clinical and pathological characteristics in PDAC patients

Clinical characteristics	Total	UBL4A mRNA expression		*P* value
		Low	High	
Age (years)				
<60	40	20	20	0.890
≥ 60	29	14	15	
Gender				
Male	49	24	25	0.940
Female	20	10	10	
TNM stage				
I + IIa	40	16	24	0.040
IIb + III	29	18	11	
Nodal metastasis				
Yes	26	17	9	0.038
No	43	17	26	
Histological differentiation				
Well	8	3	5	0.330
Moderate	37	20	17	
Poor	24	11	13	

### Transfection

Flag-tagged lentiviral vectors encoding human UBL4A (Lv-UBL4A-Flag) and empty vectors were constructed in GV341 (GeneChem, Shanghai China). The lentiviruses with scrambled shRNA against UBL4A (Lv-shUBL4A) and the shRNA control (shCtrl) were constructed in GV112 (GeneChem, Shanghai China). After lentiviral infection, single-cell clones were selected by 2.5 μg/ml puromycin (Sigma-Aldrich, St. Louis, MO, USA) for 2 to 4 weeks. The SiRNA for LAMP1 (si-LAMP1) and the siRNA negative control (si-NC) were purchased from RIBOBIO (Guangzhou, China). To induce overexpression of LAMP1, human LAMP1 (NM_005561) cDNA was cloned into a plasmid (GeneCopoeia, Guangzhou, China). For transient transfection, the cells were seeded in six well plates, and 50 nm siRNA or 2 μg plasmids were transfected into the cells using Lipofectamine 2000 (Life Technologies Limited Paisley, Grand Island, NY, USA). The efficiency of all transfections was evaluated by qRT-PCR and/or western blotting. The target sequences of the lentiviruses and siRNAs are listed in Additional file 1: Table S1.

### RNA extraction and quantitative real-time PCR analyses

Total RNA was extracted and isolated from the cell lines and frozen tumor specimens using a AxyPrep Multisource Total RNA Miniprep Kit from Axygen (Corning, Suzhou, Jiangsu, China), and the first strand cDNA was synthesized using the Rever TraAce qPCR RT Kit Master Mix with gDNA Remover (FSQ-301, Toyobo Co. Ltd. Osaka, Osaka Prefecture, Japan) according to the manufacturer’s instructions. Quantitative real-time polymerase chain reaction (qRT-PCR) was performed as previously described [10]. Briefly, qRT-PCR (SYBR Green Assay, Roche Diagnostics GmbH, Indianapolis, IN, USA) was performed on a 7500 FAST Real-Time PCR System (Applied Biosystems). The relative expression levels of the mRNA were calculated and quantified using the 2^−ΔΔT^ method after normalization to the expression of the control. GAPDH served as the endogenous control. The primer sequences are described in Additional file 2: Table S2 and were purchased from Comate Bioscience (Institute of Biotechnology, Jilin, China).

### Electron microscopy

Electron microscopy was performed as previously described [10]. Pancreatic cancer cell lines were fixed in 2.5% glutaraldehyde and postfixed in 1% osmium tetroxide buffer. Tissues were embedded in spur resin, and thin sections were cut. The sectioned grids were stained with a saturated solution of uranyl acetate and lead citrate. Sections were examined at 80 kV using a JEOL 1200EX transmission electron microscope.

### GFP-mRFP-LC3 staining

The GFP-mRFP-LC3 lentivirus was purchased from GeneChem (Shanghai China). Pancreatic cancer cell lines (CFPAC-1 and PANC-1) cultured on covers lips were transduced with the control and the GFP-mRFP-LC3 lentiviral vectoros and were then selected with puromycin (Sigma-Aldrich, St. Louis, MO, USA) for one week. Stably transfected cells were infected with the LV-UBL4A-Flag, LV-shUBL4A, LAMP1 plasmid, si-LAMP1 and their respective controls. The cells were viewed under a fluorescence microscope. Theoretically, GFP is a stably folded protein and is relatively resistant to lysosomal proteases. However, the low pH level inside the lysosomes quenches the fluorescent signal of the GFP. Therefore, autophagosomes and autolysosomes were labeled yellow (mRFP and GFP) and red (mRFP only), respectively. The numbers of GFP and mRFP dots were determined by manual counting of fluorescent puncta in five high-power fields (20×, Olympus) and analyzed using Image-Pro Plus v6.0 software. We calculated the ratio of autolysosomes (red) to autophagosomes (yellow) per cell to evaluate the extent of autophagosomes maturation into autolysosomes.

### Immunofluorescence

The tumor cells transfected with Lv-UBL4A-Flag and Lv-shUBL4A as well as their controls were seeded on 24-well plates. The cells were fixed with 4% paraformaldehyde for 30 min and were permeabilized with 0.5% Triton X-100 for 20 min. After incubation for 2 h with anti-UBL4A (Proteintech, Wuhan, Hubei, China), anti-LAMP1 (Abcam, Shanghai, China) and anti-LC3B (Cell Signaling Technology, Danvers, MA, USA), the cells were washed with PBS three times. Then, the cells were incubated with secondary antibodies for 1 h (Bioss, Beijing, China), and 4′6-diamino-2-phenylindole (DAPI, Beyotime Biotechnology, Shanghai, China) was added to stain the cell nuclei. Finally, the cells were viewed with a fluorescence microscope (20×, Olympus). Information on the primary antibodies for immunofluorescence is provided in Additional file 3: Table S3.

### Lyso-tracker red staining

Transfected cells were stained with Lyso-Tracker Red (Beyotime Biotechnology, Shanghai, China) according to the manufacturer’s instructions, and a fluorescence microscope (20×, Olympus) was used to observe fluorescence. The red signals (lysosome) were quantified and are shown in s bar graph with relevant statistics.

### Coimmunoprecipitation (co-IP)

First, 2 × 10^7^ cells were lysed with 500 μL of ice-cold polysome lysis buffer (100 mM KCl; 4 mM MgCl_2_; 10 mM HEPES, pH 7.0; 0.5% Nonidet P-40; 1 mM DTT; 100 units/ml RNase OUT; and 40 μL/mL complete protease inhibitor cocktail) for 10 min on ice. Then, the cell lysates were collected after centrifugation. UBL4A antibodies (Proteintech, Wuhan, Hubei, China) or control immunoglobulin (IgG) (Beyotime Biotechnology, Shanghai, China) were added to protein G agarose beads (EMD Millipore Corporation, Temecula, CA, USA) and allowed to bind while rotating at 4 °C overnight (for UBL4A and LAMP1) or 2 h (for IgG). The lysates were precleared with an IgG antibody and then incubated with precoated beads for 2 h at RT on a rotator. After the beads were washed, the complexes were boiled for 10 min at 100 °C and loaded on a gel for western blotting. Information on the primary antibodies for co-IP is provided in Additional file 3: Table S3.

### Orthotopic tumor model

Orthotopic tumor models were created as previously described [10]. The study protocol was approved by the Institutional Review Board of The First Affiliated Hospital of Harbin Medical University. Briefly, two luciferase-expressing cell lines (CFPAC-1-UBL4A and PANC-1-shUBL4A and their control cells) (5 × 106/0.2 mL) were injected into the right flanks of nude mice. Then, 1 mm3 pieces of tumor harvested from four mice were transplanted into four groups of mouse pancreatic tails. The animals were imaged weekly using the Night OWL II LB983 imaging system in vivo (Berthold Technologies GmbH & Co. KG, Germany). After 5 weeks of xenograft procedures, the mice were sacrificed, and the numbers of visible metastatic lesions in the gut, mesentery, liver, spleen and kidneys were recorded. The primary and metastatic pancreatic tumors were excised, weighed, and fixed in 4% paraformaldehyde.

### Statistical analysis

Statistical analysis was performed with SPSS 19.0 software or GraphPad Prism 6.01 software. The data were shown as the mean ± standard deviation (SD). Pearson analysis and Kaplan–Meier survival analysis were used to evaluate the statistical significance, and the variance between the two groups was analyzed using Student *t*-tests. Differences were considered significant when *, *P* < 0.05; **, *P* < 0.01; ***, *P* < 0.001; and non-significant when *P* > 0.05.

Details on other experimental procedures are described in the Supplementary Methods (Additional file 9: Supplementary Methods). 

## Results

### UBL4A expression decreases in PDAC, and high UBL4A expression is correlated with longer survival

**Fig. 1:**
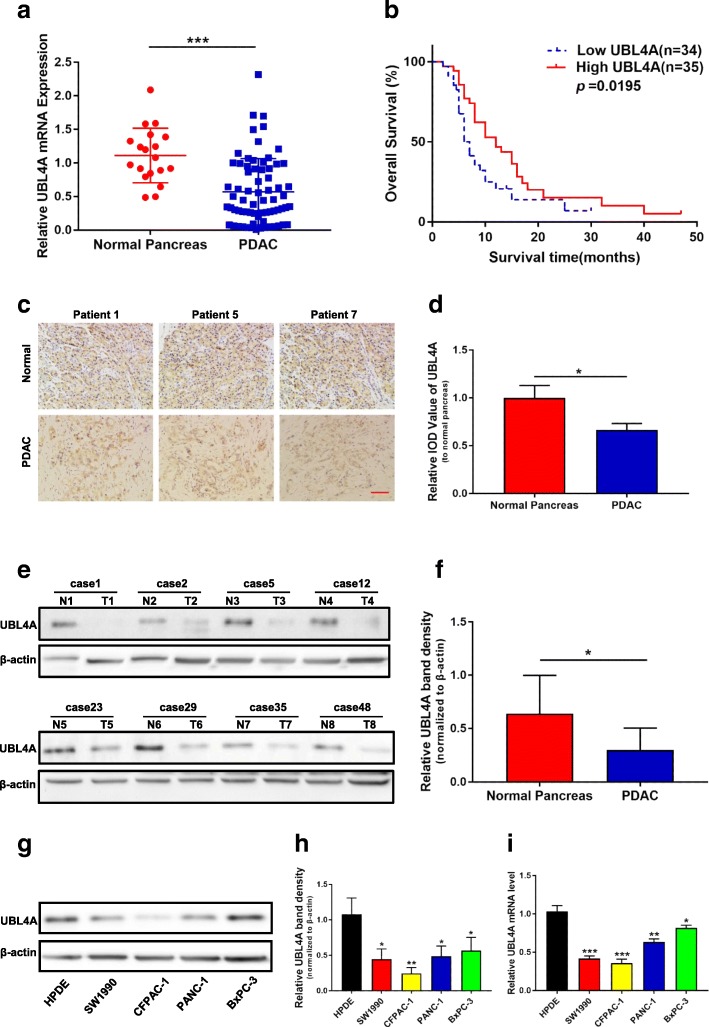
UBL4A decrease in PDAC and its high expression is correlated with longer survival. **a** The expression of UBL4A in 19 normal pancreatic tissues and 69 PDAC tissues was detected by qRT-PCR assays. **b** Kaplan–Meier plot of overall survival of patients with PDAC. The low and high levels of UBL4A expression were separated according to the median value. **c** Representative staining with antibody against UBL4A in PDAC or normal pancreatic tissues detected by immunohistochemistry (IHC) (original magnification, 20×)(bar, 400 μm). **d** Specimens were scored and estimated in relative integrated optical density (IOD) value or in percentage of positive cells. **e** Western blotting of proteins extracted from eight paired samples of tumor (T) and normal pancreatic tissues (N). **f** Densitometric quantification of western blotting results. **g, h** UBL4A protein levels in HPDE and four PDAC cell lines detected by western blotting and densitometric quantification of western blotting results. **i** Relative UBL4A mRNA levels in HPDE and four PDAC cell lines by qRT-PCR. The statistical significance between different groups was calculated with Student *t*-test. Data are shown as the mean ± SD of three replicates; *P < 0.05, **P < 0.01; ***P < 0.001; ns: not significant

Sixty-nine patients who underwent pancreatectomy for PDAC were included in this study. The expression of UBL4A in PDAC tissues was compared with that in normal tissues (18/69 patients) by qRT-PCR assays (Fig. 1a). Moreover, patients with high levels of UBL4A mRNA exhibited significantly longer overall survival than those whose tumors expressed a low UBL4A mRNA level (Fig. 1b). A clinical association analysis indicated that UBL4A expression was significantly associated with the TNM stage and lymph node metastasis in PDAC (Table 1). Moreover, compared with normal tissue samples, the level of UBL4A protein was lower in PDAC samples, as determined by immunohistochemistry (IHC) and western blot analysis (Fig. 1c-f). Regarding different cell lines, the UBL4A protein and mRNA levels were detected in HPDE cells, the human pancreatic duct epithelial cell line, and four PDAC cell lines (Fig. 1g-i). Together, all of these results demonstrate that the level of UBL4A is reduced in both PDAC tissues and cell lines and that elevated UBL4A is associated with longer survival.

### UBL4A inhibits tumor proliferation and metastasis in PDAC cell lines

**Fig. 2:**
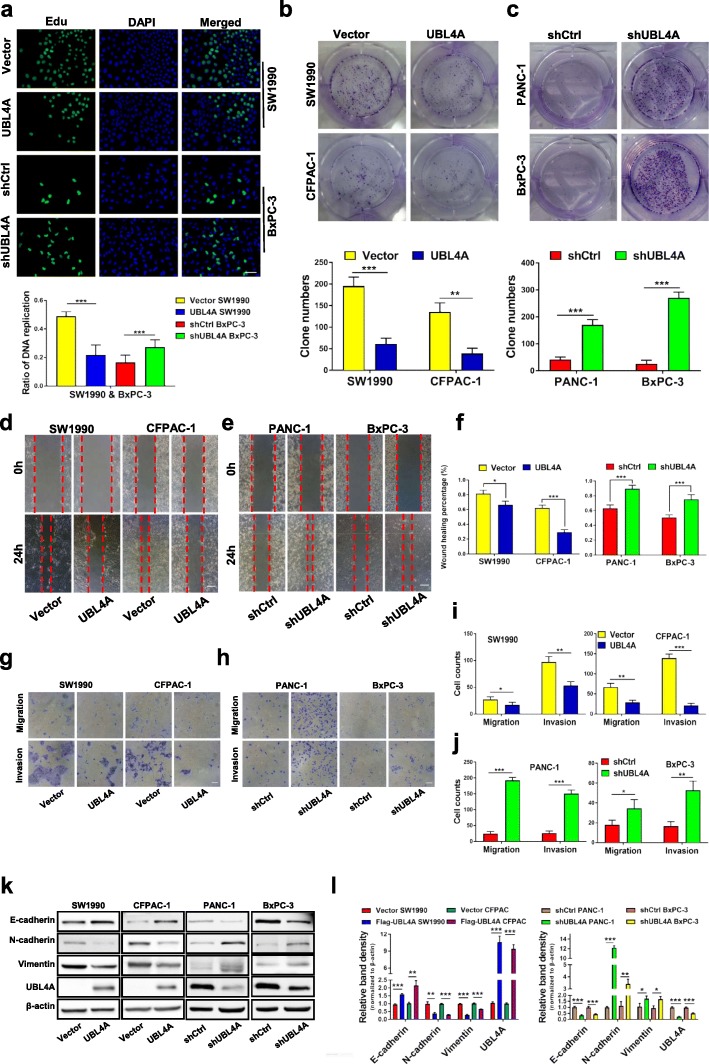
UBL4A inhibits tumor proliferation and metastasis in PDAC. **a** The proliferative capacity was determined by EdU retention assays in pancreatic cancer cells which were transfected with flag-tagged lentiviral vector encoding UBL4A (LV-UBL4A-Flag) and lentiviruses scramble shRNA against UBL4A (LV-shUBL4A) as well as their controls (original magnification, 20×) (upper panel) (bar, 50 μm) and the ratio of DNA replication in each groups were calculated (lower panel). **b, c** Representative images of colony formation assays were shown in the upper panels; the number of colonies were counted as shown in the panels below. **d-j** Transwell migration/invasion assay and wound healing assay were performed to test the effect of UBL4A on PDAC cells metastasis in vitro (original magnification, 20×) (bars, 25 μm). **k, l** The expression of E-cadherin, N-cadherin, Vimentin and UBL4A was determined by western blotting assays in four pancreatic cancer cell lines of different groups (Vector, LV-UBL4A-Flag, shCtrl, LV-shUBL4A). The statistical significance between different groups was calculated with Student *t-*test. Data are shown as the mean ± SD of three replicates; *P < 0.05, **P < 0.01; ***P < 0.001; ns: not significant

To assess the role of UBL4A in PDAC cell proliferation and metastasis, four pancreatic cancer cell lines (SW1990, CFPAC-1, PANC-1 and BxPC-3) were introduced. Through lentivirus transfection, UBL4A was effectively overexpressed in the CFPAC-1 and SW1990 cells and silenced in the PANC-1 and BxPC-3 cells. Both the overexpression and knockdown efficiency of UBL4A were confirmed by qRT-PCR and western blot assays (Additional file 4: Figure S1a-f). Cell growth assays showed that UBL4A overexpression (LV-UBL4A) suppressed cell viability and blocked the DNA replication in the indicated PDAC cells. Moreover, knockdown of UBL4A (LV-shUBL4A) in the PANC-1 and BxPC-3 cells increased their proliferation and DNA replication capability (Fig. 2a, Additional file 4: Figure S1 g-j). Consistently, colony formation assays demonstrated that the UBL4A upregulation (LV-UBL4A) groups led to the generation of fewer and smaller colonies compared with the control groups and that suppression of UBL4A (LV-shUBL4A) increased the number of colonies in the PANC-1 and BxPC-3 cells relative to the control groups (Fig. 2b-c). To assess the effect of UBL4A on the potency of the migration and invasion of pancreatic cancer cells, transwell and wound healing assays were performed. Our results demonstrated that elevated expression of UBL4A led to a significant decrease in the migratory and invasive capabilities of the CFPAC-1 and SW1990 cells. In a corresponding finding, UBL4A knockdown led to the significant promotion of cell migration and invasion in the PANC-1 and BxPC-3 cells (Fig. 2d-j). It has been widely accepted that epithelial-to-mesenchymal transition (EMT) enables tumor cells to acquire the features of high motility and invasiveness [21]. Moreover, our previous study proved that EMT progression played a crucial role in pancreatic cancer metastasis [22]. Thus, the expression of epithelial marker (E-cadherin) and mesenchymal markers (N-cadherin and vimentin) was evaluated by western blotting, and the results were consistent with those described above (Fig. 2k-l). Together, these results indicate that UBL4A inhibits tumor proliferation and metastasis in pancreatic cancer cells.

### UBL4A suppresses tumor proliferation and metastasis through inhibition of autophagy

**Fig. 3:**
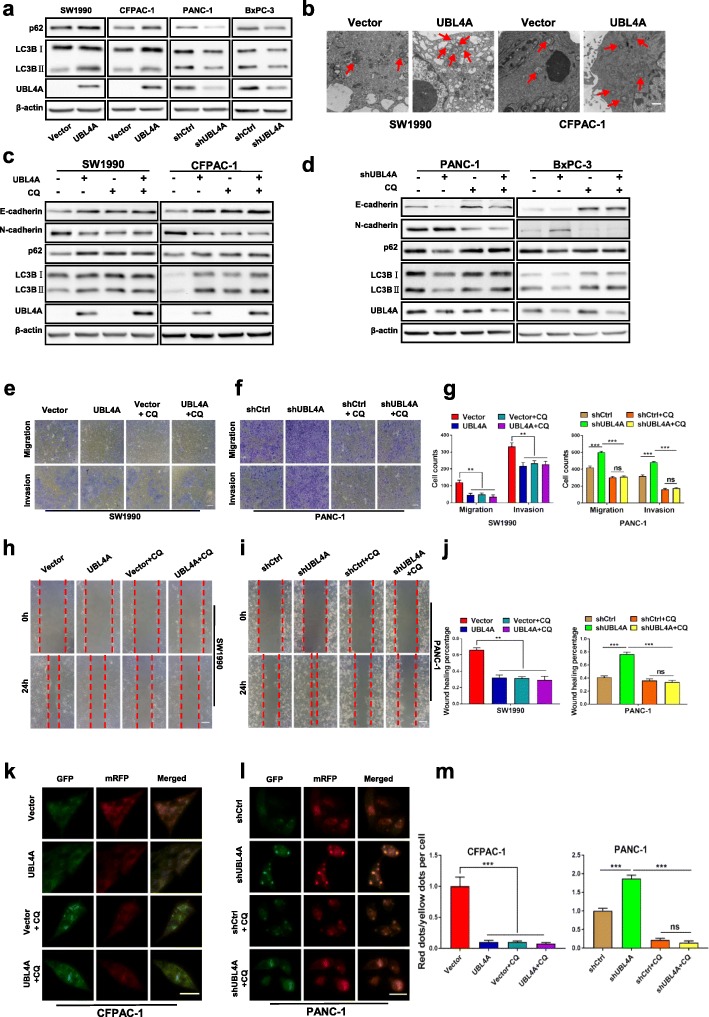
UBL4A-induced inhibition of tumor depends on autophagy. **a** Western blotting analyses of LC3B and p62 in four PDAC cell lines of different groups (Vector, LV-UBL4A-Flag, shCtrl, LV-shUBL4A). **b** Electron microscopy showed the different account of autophagosomes in SW1990 and CFPAC-1 transfected with lentiviral vector encoding UBL4A (LV-UBL4A-Flag) (bar, 100 nm). **c** Whole SW1990 and CFPAC-1 lysates in empty vector, LV-UBL4A-Flag, Chloroquine (CQ), LV-UBL4A-Flag plus CQ were subjected to western blotting to test E-cadherin, N-cadherin, p62, LC3B and UBL4A protein expression. **d** Whole PANC-1 and BxPC-3 lysates in shCtrl, shUBL4A, CQ, shUBL4A plus CQ were subjected to western blotting to test E-cadherin, N-cadherin, p62, LC3B and UBL4A protein expression. **e-g** The role of CQ in UBL4A-induced migration and invasion was demonstrated by transwell assay in SW1990 and PANC-1 (original magnification, 10×) (bars, 25 μm). **h-j** Wound healing assay was performed to detected the role of CQ in UBL4A-mediated metastasis in SW1990 and PANC-1 (original magnification, 10×) (bars, 25 μm). **k-m** The immunofluorescence assays were performed in pancreatic cancer cells that were transfected with flag-tagged mRFP-GFP-LC3 lentiviral vector in four different groups (original magnification, 20×) (bars, CFPAC-1: 25 μm, PANC-1: 10 μm). The numbers of GFP and mRFP dots were determined by fluorescent puncta in 5 high-power fields. The ratio of red dots (autolysosomes) to yellow dots (autophagosomes) per cell was calculated. The statistical significance between different groups was calculated with Student *t*-test. Data are shown as the mean ± SD of three replicates; *P < 0.05, **P < 0.01; ***P < 0.001; ns: not significant

Autophagy is important to support tumor growth in extreme environments [23]. Previous studies have demonstrated the tumorigenic roles of autophagy in pancreatic cancer [10, 24]. To determine whether autophagy is required for UBL4A-mediated tumor suppression, the correlation between autophagy and UBL4A was further tested, and the autophagy inhibitor chloroquine (CQ) was introduced to the experimental cells. LC3B (microtubule-associated protein 1 light chain 3β) plays a crucial role in functional autophagosome formation, and p62 has been suggested to act as a chaperone during the degradation of autophagosomes [25]. In addition, LC3BII, as detected by western blotting, and autophagosome formation, which is assessed by electron microscopy, have been regarded as standard markers for autophagy [14]. Our results showed that the levels of LC3BII and p62 were significantly elevated in the LV-UBL4A groups. In contrast, LV-shUBL4A decreased the LC3BII and p62 protein levels in PANC-1 and BxPC-3 cells (Fig. 3a, Additional file 5: Figure S2a-b). Consistent with the western blotting results, electron microscopy revealed a significant increase in autophagosomes in SW1990 and CFPAC-1 cells transfected with UBL4A-expressing lentiviral vectors compared with the control groups (Fig. 3b). These results indicate that UBL4A causes the accumulation of autophagosomes. In addition, UBL4A failed to induce changes in autophagy or in E-cadherin or N-cadherin expression in the presence of CQ (Fig. 3c-d, Additional file 5: Figure S2c-d). To explore whether the UBL4A inhibition of pancreatic cancer proliferation and metastasis was associated with autophagy suppression, EdU retention assays (Additional file 6: Figure S3a-c), transwell assays (Fig. 3e-g, Additional file 6: Figure S3d-f) and wound-healing assays (Fig. 3h-j, Additional file 6: FigureS3 g-i) were carried out. Consistently, CQ abolished the antitumor effects of UBL4A on tumor proliferation and metastasis in vitro. These findings consistently demonstrate that UBL4A suppresses tumor proliferation and metastasis through inhibition of autophagy, similar to the accumulation of autophagosomes.

To explore whether accelerated autophagosome synthesis or reduced autophagic vacuole maturation and degradation is responsible for UBL4A-induced autophagosome accumulation in cells, the autophagy-related genes ATG5 and ATG7, key molecules involved in autophagosome formation, were introduced [26, 27]. Western blot assays were subsequently used to analyze the expression levels of ATG5 and ATG7 among different groups. However, as shown in Additional file 7: Figure S4a, neither of these two ATGs exhibited noteworthy changes with the downregulation or upregulation of UBL4A. Thus, we speculate that UBL4A may play a role in autolysosome degradation instead of autophagosome formation. Consistently, our data showed that CQ abolished the effects of UBL4A on autophagy inhibition and tumor suppression and had no synergetic function with UBL4A (Fig. 3c-j, Additional file 6: Figure S3). These findings confirmed that UBL4A participated in regulating autolysosome degradation, which occurs during the late stage of autophagy. In addition, a tandem-labeled GFP-mRFP-LC3 reporter was used to measure autophagic flux. GFP is a stably folded protein that is resistant to lysosomal proteases. The low pH level inside lysosomes quenches the fluorescent signal of GFP. Autophagosomes and autolysosomes were labeled yellow (mRFP and GFP) and red (mRFP only), respectively [28]. As shown in Fig. 3k and m, the ratios of red dots (autolysosomes) to yellow dots (autophagosomes) per cell were significantly decreased in the UBL4A overexpressing groups compared with those in the control groups, indicating that autophagic flux was hampered after UBL4A upregulation. In contrast, suppression of UBL4A increased the ratio of red dots (autolysosomes) to yellow dots (autophagosomes) per cell in comparison with the control groups (Fig. 3l-m). Furthermore, CQ simultaneously blocked autophagic flux and eliminated the influence of UBL4A on autophagic flux (Fig. 3k-m). Together, these data indicate that UBL4A is a potent autophagic inhibitor that causes autophagosome accumulation due to impaired autophagic degradation and suppresses pancreatic cancer proliferation and metastasis by inhibiting autophagy.

### UBL4A causes lysosomal dysfunction in PDAC cells

**Fig. 4:**
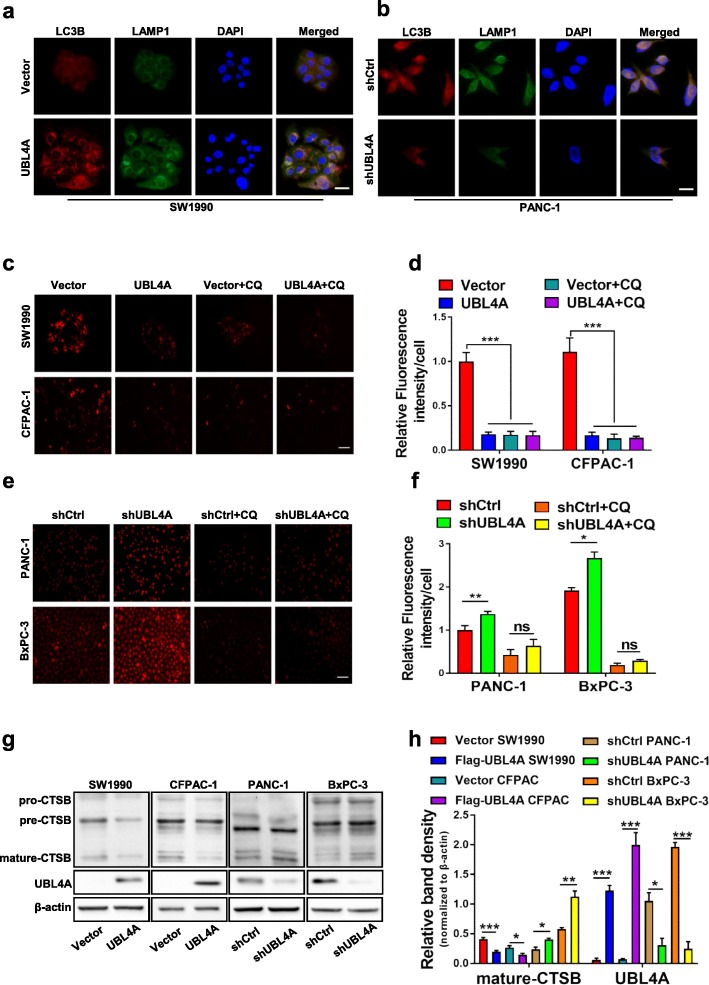
UBL4A causes lysosomal dysfunction in PDAC cells. **a, b** The immunofluorescence staining exhibited the collocation and expression of LC3B and LAMP1 in the cytoplasm in UBL4A overexpressed and downregulated groups (original magnification, 20×) (bars, 25 μm). **c-f** Lyso-Tracker Red DND-99 staining was performed on 4 PDAC cell lines of different groups (original magnification, 20×) (bars, 50 μm) and relative fluorescence intensity per cell was calculated. **g-h** Western blotting analyses of CTSB and UBL4A in four PDAC cell lines of different groups (Vector, LV-UBL4A-Flag, shCtrl, LV-shUBL4A) and relative band density were calculated. The statistical significance between different groups was calculated with Student *t*-test. Data are shown as the mean ± SD of three replicates; *P < 0.05, **P < 0.01; ***P < 0.001; ns: not significant

Impaired autophagic degradation is closely related to the immaturity of autolysosomes in that it is caused by either defective fusion between autophagosomes and lysosomes or lysosomal dysfunction [29]. To determine the exact mechanism of UBL4A-induced impaired autophagic degradation, we used fluorescence microscopy to determine the collocation of LC3B and LAMP1. Both the upregulated and downregulated UBL4A groups exhibited collocation of LC3B (red puncta) and LAMP1 (green puncta), similar to their respective control groups (Fig. 4a-b). These results indicate that UBL4A may not affect autophagosome-lysosome fusion. Thus, we next attempted to explore whether UBL4A influenced lysosomal function. Lyso-Tracker Red DND-99 is a fluorescent probe with weak alkalinity that can selectively remain in acidic lysosomes to specifically label lysosomes. Our work showed that the intensity of Lyso-Tracker Red fluorescence was lower in UBL4A overexpressing cells but higher in UBL4A-knockdown cells compared with the controls. CQ remarkably reduced the fluorescence intensity as expected in the UBL4A upregulated and downregulated groups (Fig. 4c-f). These results indicate that UBL4A causes lysosomal alkalinization in PDAC cells. Moreover, lysosomal degradation depends on the amount and activity of hydrolases. Cathepsins are the most studied lysosomal hydrolases that participate in autophagic and lysosomal degradation [30], and the relationship between UBL4A and cathepsins is still unknown. We then investigated whether UBL4A affects the expression and enzymatic activity of cathepsin B (CTSB). Reduced expression of CTSB was observed at the protein level and primarily involved the mature forms of the protein in UBL4A overexpressing cells compared with the control groups. Consistently, UBL4A down-regulation in PANC-1 and BxPC-3 cells increased the activity of proteases compared to the control groups (Fig. 4g-h). Together, these data verify that UBL4A causes lysosomal dysfunction due to simultaneous lysosomal alkalinization and inhibition of the expression and maturation of CTSB. Moreover, the impaired autophagic degradation caused by UBL4A is attributed to lysosomal dysfunction rather than impaired fusion between autophagosomes and lysosomes.

### UBL4A directly targets LAMP1 in pancreatic cancer

**Fig. 5:**
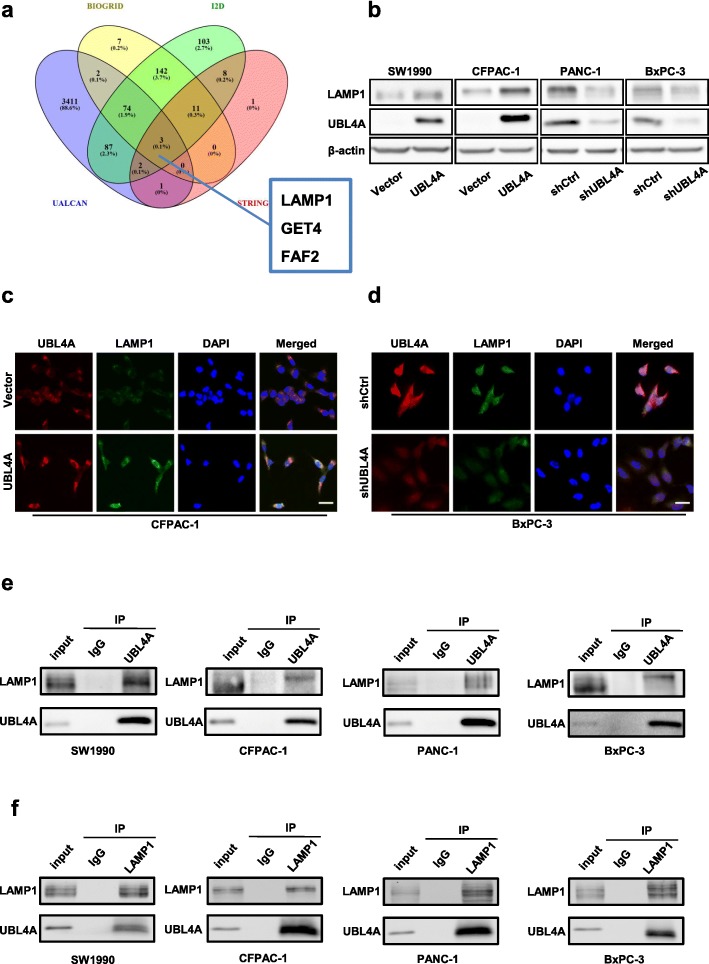
UBL4A directly targets LAMP1 in PDAC. **a** Intersections among four online protein interaction databases. **b** Western blotting analyses of LAMP1 and UBL4A in four PDAC cell lines of different groups (Vector, LV-UBL4A-Flag, shCtrl, LV-shUBL4A). **c-d** The immunofluorescence staining exhibited the collocation and expression of UBL4A and LAMP1 in the cytoplasm in UBL4A overexpressed and downregulated groups (original magnification, 20×) (bars, 25 μm). **e** UBL4A was immunoprecipitated from cell lysates of four PDAC cells and LAMP1 was detected using LAMP1 antibody. **f** LAMP1 was immunoprecipitated from cell lysates of four PDAC cells and UBL4A was detected using UBL4A antibody. The statistical significance between different groups was calculated with Student *t-*test. Data are shown as the mean ± SD of three replicates; *P < 0.05, **P < 0.01; ***P < 0.001; ns: not significant

To identify the putative downstream target of UBL4A, four online interactive protein databases, BIOGRID version 3.5.168, I2D (Interologous Interaction Database) version 2.9 [31], UALCAN [32] and STRING version 10.5, were used simultaneously. LAMP1, GET4 and FAF2 were found in all four databases (Fig. 5a). We focused on LAMP1 because of its involvement in lysosomal exocytosis, in movement of lysosomes along microtubules, and in the fusion of phagosomes with lysosomes [33]; it may also play a role in tumor cell metastasis [34]. Furthermore, LAMP1, as a lysosomal marker, had already been detected by immunofluorescence in our study (Fig. 4a, b), and its expression was positively correlated with UBL4A. To validate the potential correlation, the mRNA expression of UBL4A and LAMP1 in 69 PDAC samples was quantitatively analyzed by qRT-PCR. As shown in Additional file 7: Figure S4b, the LAMP1 mRNA levels were positively related to the expression of UBL4A in PDAC tissues (r^2^ = 0.2879, *P* < 0.001). Western blotting showed that UBL4A inhibition significantly reduced the LAMP1 protein level and that increased UBL4A remarkably increased LAMP1 protein expression compared to their respective control groups (Fig. 5b, Additional file 7: Figure S4e). In addition, we found that UBL4A inhibition remarkably decreased the LAMP1 mRNA levels, and increased UBL4A dramatically upregulated LAMP1 mRNA expression compared to the control groups (Additional file 7: Figure S4c-d). For additional analyses, immunofluorescence (IF) staining was performed. UBL4A-overexpressing cells enhanced the expression of LAMP1, and suppression of UBL4A reduced the fluorescence intensity of LAMP1 (Fig. 5c-d). In addition, the collocation of UBL4A and LAMP1 was shown in IF staining assays, providing rational evidence for their interaction in the cytoplasm (Fig. 5c-d). To ascertain whether UBL4A directly interacts with LAMP1 in pancreatic cancer cells, UBL4A and LAMP1 were immunoprecipitated from cell lysates of the pancreatic cancer cells. As shown in Fig. 5e-f, the specific, direct interaction between UBL4A and LAMP1 was confirmed by a coimmunoprecipitation (co-IP) assay. These results indicate that UBL4A directly targets LAMP1 in PDAC.

### LAMP1 is involved in UBL4A-mediated inhibition of autophagy and antitumor effects

**Fig. 6:**
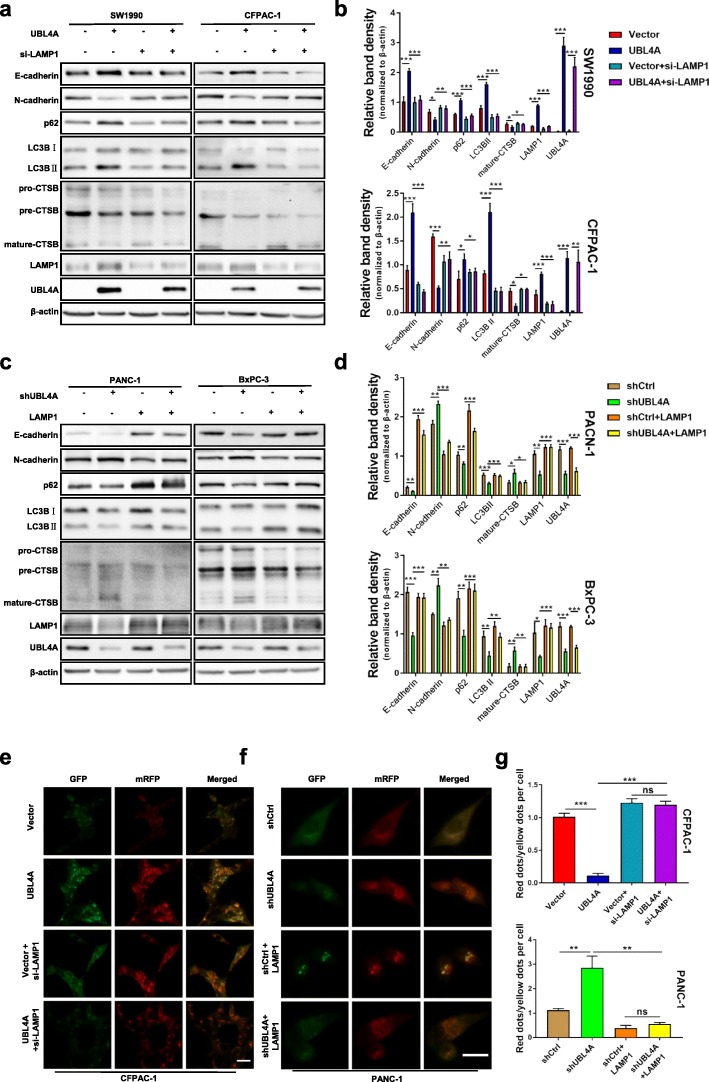
LAMP1 involves in UBL4A-mediated inhibition of autophagy. **a, b** SiLAMP1 was transiently transfected in UBL4A overexpressed cell lines (SW1990, CFPAC-1) and forty-eight hours after transfection, the cell lysates were subjected to western blotting. **c, d** LAMP1 plasmid was transiently transfected in UBL4A downregulated cell lines (PANC-1, BxPC-3) and the cell lysates were subjected to western blotting. **e** Representative fluorescent photographs of autophagy flux assay in mRFP-GFP-LC3 tagged CFPAC-1 were subjected to Vector, LV-UBL4A-Flag, siLAMP1, LV-UBL4A-Flag +siLAMP1 groups (original magnification, 20×) (bar, 200 μm). **f** Representative fluorescent photographs of autophagy flux assay in mRFP-GFP-LC3 tagged PANC-1 were subjected to control, lv-shUBL4A, LAMP1 plasmid and lv-shUBL4A + LAMP1 plasmid groups (original magnification, 20×) (bar, 20 μm). **g** The numbers of GFP and mRFP dots were determined by fluorescent puncta in 5 high-power fields The ratio of red dots (autolysosomes) to yellow dots (autophagosomes) per cell was calculated. The statistical significance between different groups was calculated with Student *t*-test. Data are shown as the mean ± SD of three replicates; *P < 0.05, **P < 0.01; ***P < 0.001; ns: not significant

**Fig. 7:**
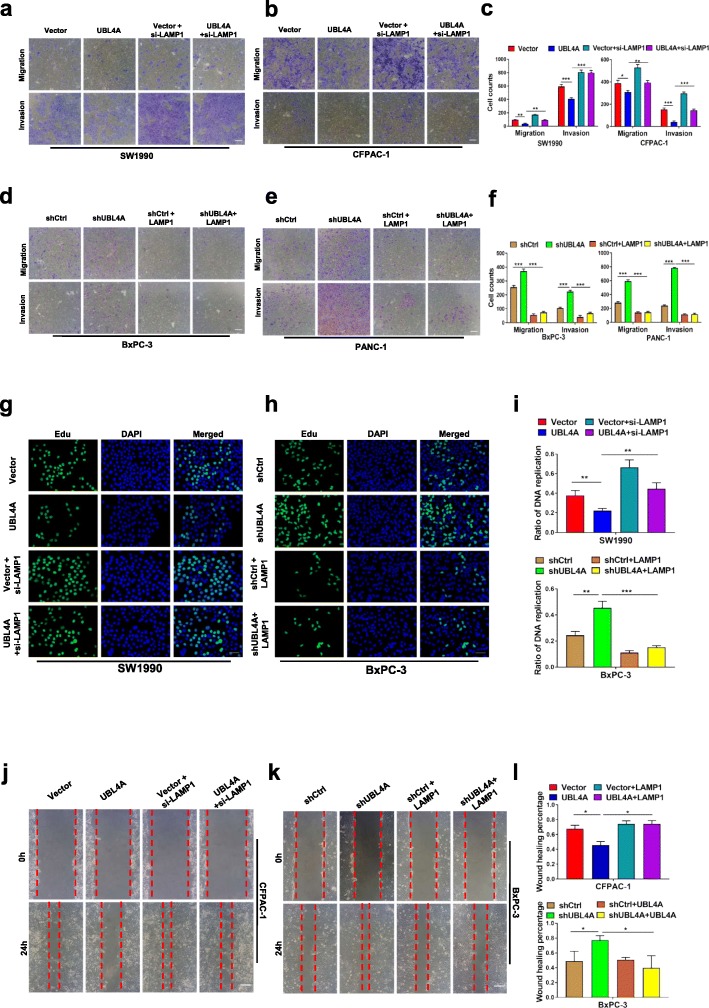
LAMP1 involves in UBL4A-mediated anti-tumor effects. **a-f** The role of LAMP1 in UBL4A-induced migration and invasion was demonstrated by transwell assay in four PDAC cell lines (original magnification, 10×) (bars, 25 μm). **g** The proliferative capacity of SW1990 was determined by EdU retention assays (original magnification, 20×) (bars, 50 μm) in four different groups (Vector, lv-UBL4A-Flag, siLAMP1 and lv-UBL4A-Flag+siLAMP1). **h** Representative fluorescent photographs of Edu retention assay in BxPC-3 were subjected to shCtrl, LV-shUBL4A, LAMP1 plasmid and LV-shUBL4A + LAMP1 plasmid groups. **i** The ratio of DNA replication was calculated. **j-l** Wound healing assay was performed to detected the role of LAMP1 in UBL4A-mediated metastasis in CFPAC-1 and BxPC-3 (original magnification, 10×) (bars, 25 μm). The statistical significance between different groups was calculated with Student *t*- test. Data are shown as the mean ± SD of three replicates; *P < 0.05, **P < 0.01; ***P < 0.001; ns: not significant

To verify the role of LAMP1 in UBL4A-induced tumor suppression and inhibition of autophagy, a siRNA against LAMP1 and a LAMP1 overexpression plasmid were introduced. The silencing and overexpressing efficiencies were confirmed by western blotting and qRT-PCR. The expression of LAMP1 was notably decreased by si-LAMP1#2, and si-LAMP1#2 was chosen for further experiments (Additional file 8: Figure S5a-d). Knockdown of LAMP1 reversed the UBL4A-induced autophagy suppression, mesenchymal-to-epithelial transition (Fig. 6a-b), inhibiton of autophagic flux (Fig. 6e), inhibition of cancer cell migration and invasion (Fig. 7a-c), cell proliferation (Fig. 7g) and wound healing capacity (Fig. 7j). Consistently, LAMP1 overexpression reversed the stimulation of autophagy in UBL4A-knockdown cells (Fig. 6c-d). CTSB and N-cadherin levels (Fig. 6c-d), autophagic flux activation (Fig. 6f-g), cell migration and invasion (Fig. 7d-f), DNA replication activity (Fig. 7h) and wound healing ability (Fig. 7k-l) were also reversed by LAMP1 overexpression in UBL4A-knockdown cells. Together, these data indicate that LAMP1 restoration in pancreatic cancer cells reverses the UBL4A-induced antitumor effects and inhibition of autophagy. In summary, these results demonstrate that UBL4A inhibits autophagy-mediated proliferation and metastasis of pancreatic ductal adenocarcinoma by directly targeting LAMP1.

### UBL4A inhibits tumor proliferation and metastasis in an orthotopic tumor model

**Fig. 8:**
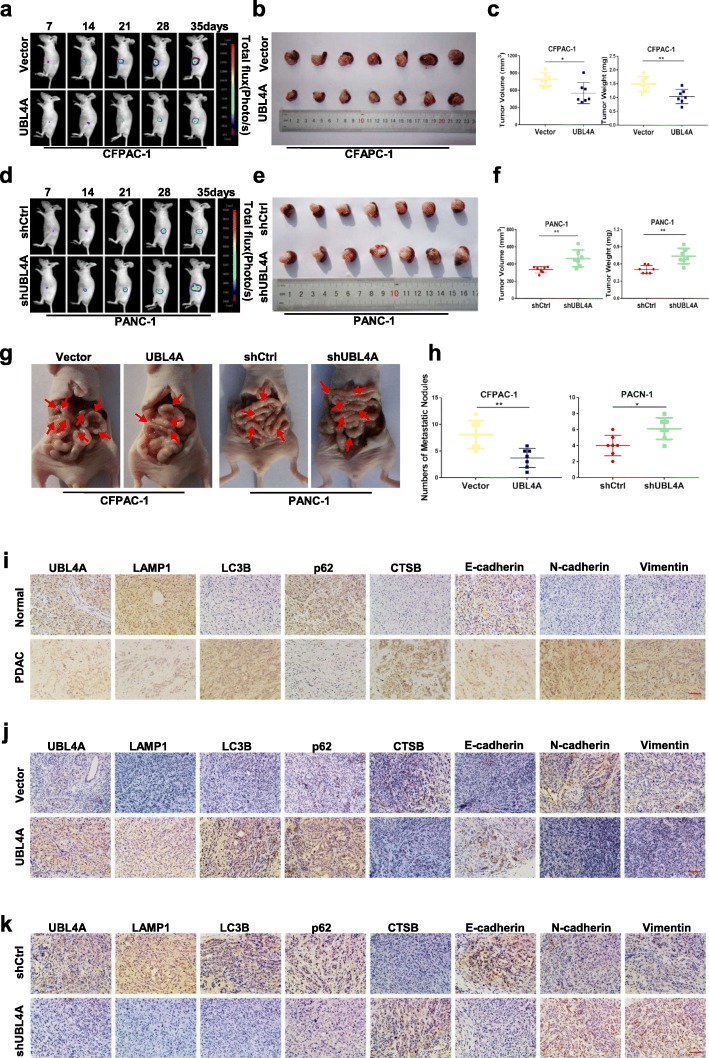
UBL4A inhibits tumor proliferation and metastasis in orthotopic tumor mode. **a-f** Representative bioluminescence imaging (following intraperitoneal injection of 0.1 mg/g luciferin) of mice at the day of 7, 14, 21, 28, and 35. At day 35, all mice were sacrificed and the primary tumors were removed, the tumor volume and weight was evaluated. **g-h** Representative images of orthotopic xenograft pancreatic cancer mouse models from four groups and metastatic nodes were calculated, red arrows indicated metastatic lesions. **i** The expression of UBL4A, LAMP1, LC3B, p62, CTSB, E-cadherin, N-cadherin and vimentin were analyzed by immunohistochemistry in human PDAC tissues and normal pancreas (original magnification, 20×) (bar, 400 μm) **j-k** The expression of UBL4A, LAMP1, LC3B, p62, CTSB, E-cadherin, N-cadherin and vimentin were analyzed in paraffin-embedded tissue sections of orthotopic pancreatic cancer models from different groups by immunohistochemistry (original magnification, 20×) (bars, 400 μm). The statistical significance between different groups was calculated with Student *t*-test. Data are shown as the mean ± SD of three replicates; *P < 0.05, **P < 0.01; ***P < 0.001; ns: not significant

To assess the role of UBL4A on PDAC proliferation and metastasis in vivo, stably transfected cell lines (CFPAC-1-Vector, CFPAC-1-UBL4A, PANC-1-shCtrl and PANC-1-shUBL4A) were used to develop a pancreatic orthotopic tumor model. The animals were imaged weekly, and the sizes of the tumors were recorded at day 35. As shown in Fig. 8a, compared with the control groups, the average size of tumors in the UBL4A-overexpression group increased relatively slowly. High UBL4A expression induced a small tumor size and reduced tumor weight in the orthotopic tumor model (Fig. 8b-c). In contrast, larger and heavier pancreatic orthotopic tumors were observed in the UBL4A-knockdown groups relative to the controls (Fig. 8d-f). Furthermore, fewer visible metastatic nodes were found in the gut of mice administered cells of the UBL4A-overexpression groups compared with those administered the control groups. In contrast, knockdown of UBL4A promoted metastatic node formation in the gut compared with the controls (Fig. 8g-h). Additionally, immunohistochemical results showed that the UBL4A level was positively correlated with the expression of LAMP1, LC3B, p62, and E-cadherin and negatively correlated with CTSB, vimentin, and N-cadherin in pancreatic cancer patients (Fig. 8i). Moreover, increased expression of UBL4A caused increased levels of LAMP1, LC3B, p62, and E-cadherin and resulted in decreased levels of CTSB, vimentin, and N-cadherin in the orthotopic pancreatic cancer models (Fig. 8j). In contrast, the opposite effects were observed in the UBL4A-knockdown groups (Fig. 8k). Taken together, our data suggest that UBL4A inhibits tumor proliferation and metastasis in orthotopic pancreatic cancer models.

## Discussion

Autophagy is a dynamic and continuous process involving the formation of autophagosomes (early stage) and lysosomal degradation after fusion (late stage [35]. Autophagy is regarded as a double-edged sword [36], and its role in cancer is context-dependent and tumor stage-dependent [37]. J Nassour et al. suggested that autophagy is an integral component of the tumor suppressive crisis mechanism and that loss of autophagy is required for the initiation of cancer [38]. However, our previous study showed that elevated autophagy was positively associated with tumor progression in PDAC tissues and cell lines [10]. During the early stages of cancer, autophagy may inhibit tumor initiation by restricting tumor necrosis and inflammatory cell infiltration. However, autophagy tends to act as a promoter of metastasis by enhancing metastatic cell survival and colonization in distant sites during the advanced stages of cancer. Our study showed that an elevated autophagic level was associated with high proliferation and metastasis of PDAC and that CQ exerted a prominent antitumor effect in pancreatic cancer (Fig. 3). Due to the understanding of the prominent role of autophagy in late-stage carcinogenesis, autophagy inhibition has emerged as an appealing therapeutic strategy in pancreatic cancer [39, 40].

UBL4A is a small ubiquitin-like protein encoded by a housekeeping gene [4]. As part of a cytosolic protein quality control complex, the BAG6/BAT3 complex, UBL4A is an essential protein that functions in the protein degradation of defective polypeptides and tail-anchored transmembrane protein biogenesis and delivery [5, 41]. In addition, UBL4A represses tumorigenesis through dephosphorylation of STAT3 [7] and is involved in DNA-damage-induced cell death [42]. Previous studies demonstrated that UBL4A is critical for the migration and the innate immune responses of fibroblasts and macrophages [43, 44]. Moreover, UBL4A interacted with actin-related protein (Arp2/3) and promoted actin branches, which served as “bridges” that guide Akt to the plasma membrane for activation [45]. All these previous studies indicate that this protein is versatile. However, the underlying molecular mechanisms by which UBL4A regulates autophagy and contributes to pancreatic cancer development and progression remain unknown.

In this study, we observed that UBL4A inhibited tumor proliferation and metastasis through the suppression of autophagy. To explore the mechanisms of UBL4A, we showed that overexpression of UBL4A promoted the accumulation of autophagosomes, possibly due to either accelerated autophagosome synthesis or impaired autophagic vacuole maturation and degradation. The autophagy-related genes ATG5 and ATG7 were initially detected. Both of these genes appear to be specifically involved in autophagosome formation [46]. Our study revealed that UBL4A-mediated autophagy inhibition and autophagosome accumulation are not required for accelerated autophagosome synthesis. As a result, we speculated that UBL4A impaired autophagy during the late stage. Next, CQ, which blocks late-stage autophagy, was used to examine the effects of UBL4A on autophagosome processes and the relationship between autophagy and tumor progression. Intriguingly, CQ eliminated the influence of UBL4A on autophagic flux but could not cooperate with UBL4A during the inhibition of tumor proliferation and metastasis, which indicated that UBL4A was a potent autophagic inhibitor that caused impaired autophagic degradation. We also found that UBL4A caused lysosomal dysfunction rather than impaired fusion between autophagosomes and lysosomes by verifying the collocation of LC3B and LAMP1. Functional lysosomes have the unique feature of having a highly acidic pH, and lysosomal degradation depends on the concentration and activity of the hydrolases, such as cathepsin B [47]. Previous studies have shown that lysosomotropic compounds exert a suppressive effect downstream of autophagosome formation by inhibiting autophagosome and lysosome fusion and/or blocking the degradation of the autophagic cargo inside autolysosomes [48, 49]. Similarly, our study describes an exact molecular mechanism that regulates late-stage autophagy, particularly lysosomal degradation, which may represent a useful adjuvant in antitumor therapy.

After confirming the role of UBL4A in autophagy inhibition, we further demonstrated that LAMP1 was a direct target of UBL4A. LAMP1 is distributed among autophagic and endolysosomal organelles and is routinely used as a lysosomal marker, and LAMP1-positive organelles are often referred to as lysosomal compartments. Despite its abundance, the role of LAMP1 in autophagy is ambiguous and insignificant according to previous studies [20]. Recently, Cheng et al. demonstrated that a significant portion of LAMP1-labeled organelles lack major lysosomal hydrolases. Their data called for caution in interpretation: LAMP1-labeled organelles do not necessarily represent degradative lysosomes or autolysosomes [50, 51]. Similarly, our study demonstrated that the restoration of LAMP1 abolished UBL4A-knockdown induced autophagy activation and influenced the expression and maturation of lysosomal hydrolases. Although the exact molecular mechanism is not clear, LAMP1 actually had an impact on regulating the expression and activity of lysosomal hydrolases, at least in UBL4A-mediated inhibition of autophagy. LAMP2, which is 37% identical to LAMP1, is essential for the degradation of the autophagosomal content via the proper fusion of lysosomes with autophagosomes in the last stage of autophagic flux [20]. To explore whether LAMP2 participates in UBL4A-mediated inhibition of autophagy, we investigated the expression of LAMP2 in UBL4A downregulated and upregulated groups by western blotting. Our results provide no evidence that UBL4A could regulate LAMP2, at least in pancreatic cancer (Additional file 8: Figure S5e). Together, the above results comprehensively demonstrate that LAMP1 plays a crucial role in UBL4A-induced autophagy inhibition, and they provide novel insights regarding LAMP1 in autophagy regulation.

Our findings suggest a new mechanism for the regulation of proliferation and metastasis by the UBL4A/LAMP1/autophagy axis in pancreatic cancer and reveal a direct interaction between UBL4A and LAMP1. However, in this study, we did not determine whether UBL4A regulates the expression of LAMP1through an exact mechanism, such as the ubiquitination–proteasome pathway, or which signaling pathways contribute to the UBL4A-induced antitumor effects in pancreatic cancers. Nevertheless, our data indicate that UBL4A suppresses pancreatic cancer development by directly regulating LAMP1.

## Conclusion

**Fig. 9:**
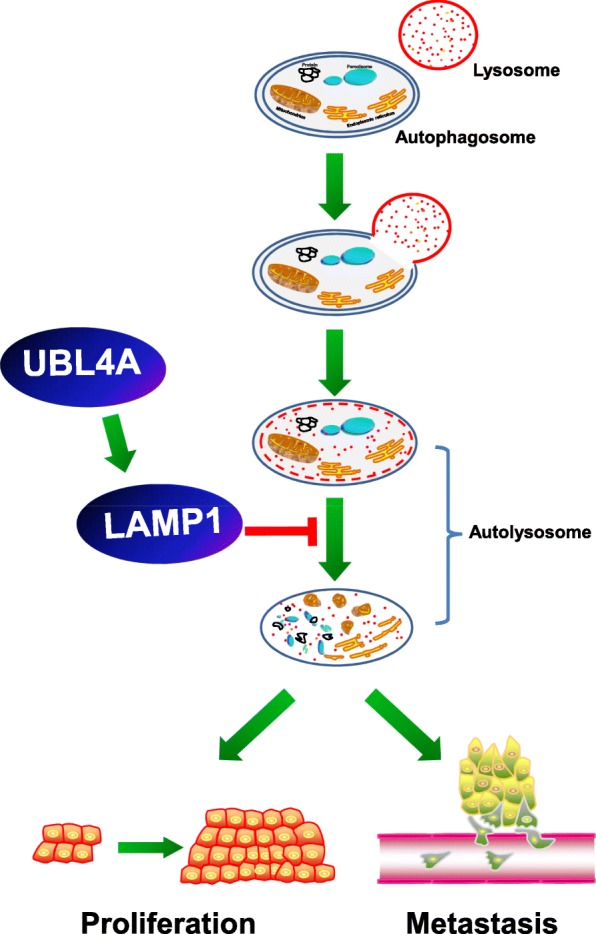
Graphical illustration of the signaling mechanisms underlying UBL4A-mediated PDAC proliferation and metastasis inhibition. UBL4A impairs the degradation processes in the autolysosome and inhibits the proliferation and metastasis of the pancreatic cancer. LAMP1 is involved in UBL4A-induced tumor suppression and inhibition of autophagy

In summary, our data highlight that UBL4A is a novel autophagy inhibitor and tumor suppressor in PDAC. Mechanistically, UBL4A is involved in late stage autophagy, particularly in lysosomal dysfunction, causing impaired degradation of autophagosomes. In addition, we demonstrate that LAMP1 is a direct target in UBL4A-induced tumor suppression and inhibition of autophagy. The current data provide greater insights into the function of the UBL4A/LAMP1 autophagy axis in the aggressive progression of pancreatic cancer (Fig. 9). Our findings demonstrate the valuable function of UBL4A, which may perform as a novel therapeutic target in the treatment of pancreatic cancer.

## Additional files

Additional file 1: **Table S1.** The target sequences of lentiviruses and siRNAs used in transfection. (DOCX 14 kb)

Additional file 2: **Table S2.** Sequences of primers used in Real-time RT-PCR. (DOCX 13 kb)

Additional file 3: **Table S3.** Primary antibodies for WB, IHC, IF and co-IP. (DOC 38 kb)

Additional file 4: **Figure S1.** The efficiency of UBL4A downregulation and upregulation is analyzed and UBL4A inhibits tumor proliferation in PDAC. **a, b** The efficiency of UBL4A overexpression (LV-UBL4A-flag) in SW1990 and CFPAC-1 was detected by western blotting. **c, d** The knockdown efficiency of LV-shUBL4A in PANC-1 and BxPC-3 was analyzed by western blotting. **e, f** The efficiency of UBL4A downregulation and upregulation was analyzed by qRT-PCR in four PDAC cell lines. **g-j** Proliferation rate was analyzed by CCK-8 assay of indicated SW1990, CFPAC-1 (Vector, LV-UBL4A) and PANC-1, BxPC-3 (shCtrl, LV-shUBL4A). The statistical significance between different groups was calculated with Student *t*-test. Data are shown as the mean ± SD of three replicates; *P < 0.05, **P < 0.01; ***P < 0.001; ns: not significant. (PDF 291 kb)

Additional file 5: **Figure S2.** The relative band density of results in western blotting. **a, b** The expression of LC3B and p62 in four PDAC cell lines of different groups (Vector, LV-UBL4A-Flag, shCtrl, LV-shUBL4A) were calculated. **c** The relative band density of E-cadherin, N-cadherin, p62, LC3B and UBL4A in SW1990 and CFPAC in four different groups. **d** The relative band density of E-cadherin, N-cadherin, p62, LC3B and UBL4A in PANC-1 and BxPC-3 in four different groups. The statistical significance between different groups was calculated with Student *t*-test. Data are shown as the mean ± SD of three replicates; *P < 0.05, **P < 0.01; ***P < 0.001; ns: not significant. (PDF 334 kb)

Additional file 6: **Figure S3.** UBL4A-induced inhibition of tumor depends on autophagy. **a-c** The proliferative capacity of pancreatic cancer cells was determined by EdU retention assays (original magnification, 20×) (bars, 50 μm) and the ratio of DNA replication was calculated. **d-f** The role of CQ in UBL4A-induced migration and invasion was demonstrated by transwell assay in CFPAC-1 and BxPC-3 (original magnification, 10×) (bars, 25 μm). **g-i** Wound healing assay was performed to detected the role of CQ in UBL4A-mediated metastasis in CFPAC-1 and BxPC-3 (original magnification, 10×) (bars, 25 μm). The statistical significance between different groups was calculated with Student *t*-test. Data are shown as the mean ± SD of three replicates; *P < 0.05, **P < 0.01; ***P < 0.001; ns: not significant. (PDF 259 kb)

Additional file 7: **Figure S4.** UBL4A-induced Autophagy inhibition is related with LAMP1 rather than ATG5 or ATG7. **a** The expression of ATG5 and ATG7 in CFPAC-1 and BxPC-3 of different groups (Vector, LV-UBL4A-Flag, shCtrl, LV-shUBL4A) were analyzed by western blotting. **b** qRT-PCR assays examined the expression of both UBL4A and LC3B mRNA in each of 69 PDAC tissues, and the relevance was listed in each blot (r^2^ = 0.2879, *P* < 0.0001). **c-d** qRT-PCR analyses of LAMP1 in four PDAC cell lines of different groups (Vector, LV-UBL4A-Flag, shCtrl, LV-shUBL4A). **e** The expressions of LAMP1 and UBL4A in four PDAC cell lines of different groups (Vector, LV-UBL4A-Flag, shCtrl, LV-shUBL4A) were calculated. The statistical significance between different groups was calculated with Student *t-*test. Data are shown as the mean ± SD of three replicates; *P < 0.05, **P < 0.01; ***P < 0.001; ns: not significant. (PDF 279 kb)

Additional file 8: **Figure S5.** The efficiency of LAMP1 downregulation and upregulation is detected by western blot and qRT-PCR. **a-b** The efficiency of LAMP1 downregulation and upregulation was detected by western blotting. **c-d** The efficiency of LAMP1 downregulation and upregulation was detected by qRT-PCR. **e** The expression of LAMP2 in CFPAC-1 and BxPC-3 of different groups (Vector, LV-UBL4A-Flag, shCtrl, LV-shUBL4A) were analyzed by western blotting. The statistical significance between different groups was calculated with Student *t*-test. Data are shown as the mean ± SD of three replicates; *P < 0.05, **P < 0.01; ***P < 0.001; ns: not significant. (PDF 210 kb)

Additional file 9: Supplementary Methods. (DOCX 25 kb)

## Data Availability

All data generated or analyzed during this study are included in this published article and its supplementary information files.

## Abbreviations

ATG: Autophagy-related gene
CQ: Chloroquine
CTSB: Cathepsin B
EMT: Epithelial-mesenchymal transition
GFP: Green fluorescent protein
IHC: Immunohistochemistry
LAMP1: Lysosome associated membrane protein-1
LAMP2: Lysosome associated membrane protein-2
LC3B: Microtubule-associated protein 1 light chain 3 beta
LV: Lentivirus
PDAC: Pancreatic ductal adenocarcinoma
qRT-PCR: Real-time quantitative reverse transcription-polymerase chain reaction
RFP: Red fluorescent protein
UBL4A: Ubiquitin-like protein 4A
