# Correction: UBL4A inhibits autophagy-mediated proliferation and metastasis of pancreatic ductal adenocarcinoma via targeting LAMP1

**DOI:** 10.1186/s13046-026-03696-5

**Published:** 2026-03-25

**Authors:** Hongze Chen, Le Li, Jisheng Hu, Zhongjie Zhao, Liang Ji, Chundong Cheng, Guangquan Zhang, Tao Zhang, Yilong Li, Hua Chen, Shangha Pan, Bei Sun

**Affiliations:** 1 Department of Pancreatic and Biliary Surgery, The First Affiliated Hospital of Harbin Medical University, 23 Youzheng Street, Nangang District, Harbin, 150001 Heilongjiang Province China; 2 Laboratory of Hepatosplenic Surgery, Ministry of Education, Harbin, Heilongjiang China; 3 Department of Breast Surgery, The First Affiliated Hospital of Harbin Medical University, Harbin, Heilongjiang China

**Correction: J Exp Clin Cancer Res 38**,** 297 (2019)**

**https://doi.org/10.1186/s13046-019-1278-9**

Following publication of the original article [1], the authors found errors in Figs. 1 and 7, and 8. Specifically:

- Figure 1c (Immunohistochemistry) - In the “Normal Pancreas” panel, the image for Patient 7 (P7) was found to be a duplicate of the image for Patient 1 (P1). This was an unintentional layout error. During the figure assembly process, the representative image for Patient 1 was accidentally pasted into the position designated for Patient 7.
- Figure 7k and Figure S3h (Wound Healing Assay) - The wound healing image (24 h) for the shCtrl+LAMP1 group in Fig. 7k duplicates the image for the shUBL4A + CQ (24 h) group in Figure S3h. This error arose from a file naming confusion.
- Fig. 7a and Figure 3e (Migration Assay) - The Vector migration image in Fig. 7a duplicates the Vector migration image in Fig. 3e.
- Figure 8k (Immunohistochemistry) - In the shUBL4A group, the image displayed for N-cadherin staining is identical to the image for Vimentin staining.
- Fig. 8k and j (Control Groups) - The N-cadherin image in the shCtrl group (Fig. 8k) was found to be a duplicate of the N-cadherin image in the Vector group (Fig. 8j).

The correct figures are provided below:

**Incorrect** Fig. 1.

**Fig. 1:**
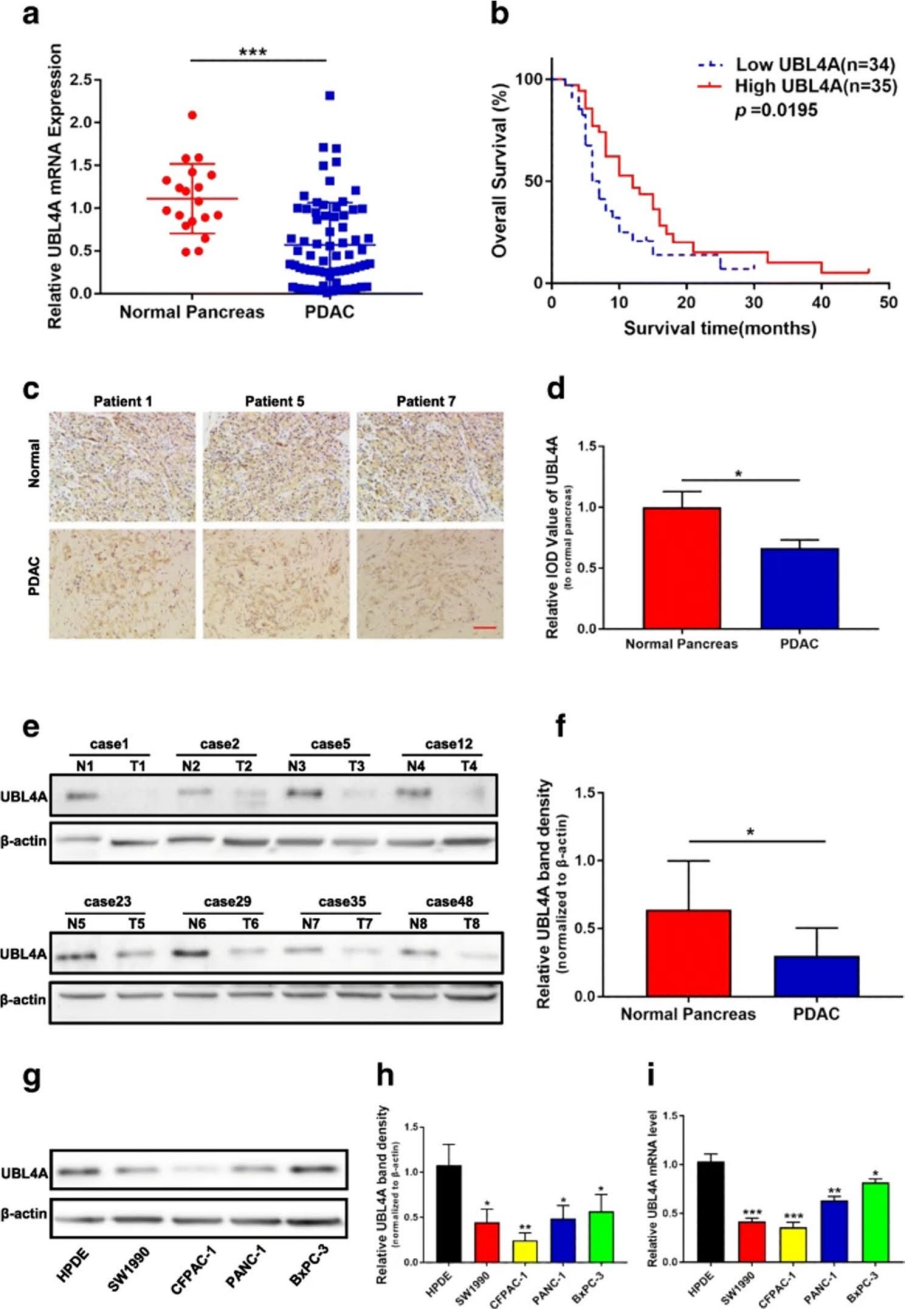
UBL4A decrease in PDAC and its high expression is correlated with longer survival. **a** The expression of UBL4A in 19 normal pancreatic tissues and 69 PDAC tissues was detected by qRT-PCR assays. **b** Kaplan–Meier plot of overall survival of patients with PDAC. The low and high levels of UBL4A expression were separated according to the median value. **c** Representative staining with antibody against UBL4A in PDAC or normal pancreatic tissues detected by immunohistochemistry (IHC) (original magnification, 20×)(bar, 400 μm). **d** Specimens were scored and estimated in relative integrated optical density (IOD) value or in percentage of positive cells. **e** Western blotting of proteins extracted from eight paired samples of tumor (T) and normal pancreatic tissues (N). **f** Densitometric quantification of western blotting results. **g**,** h** UBL4A protein levels in HPDE and four PDAC cell lines detected by western blotting and densitometric quantification of western blotting results. **i** Relative. UBL4A mRNA levels in HPDE and four PDAC cell lines by qRT-PCR. The statistical significance between different groups was calculated with Student t-test. Data are shown as the mean ± SD of three replicates; *P < 0.05, **P < 0.01; ***P < 0.001; ns: not significant

**Correct** Fig. 1.

**Fig. 1:**
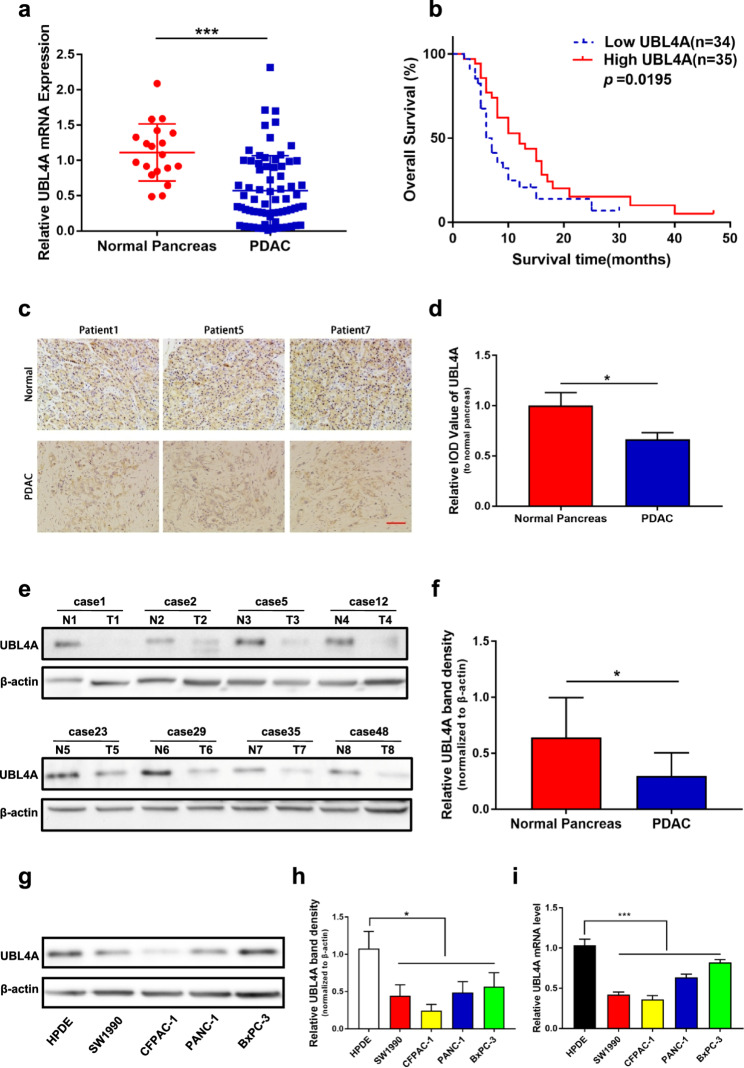
UBL4A decrease in PDAC and its high expression is correlated with longer survival. **a** The expression of UBL4A in 19 normal pancreatic tissues and 69 PDAC tissues was detected by qRT-PCR assays. **b** Kaplan–Meier plot of overall survival of patients with PDAC. The low and high levels of UBL4A expression were separated according to the median value. **c** Representative staining with antibody against UBL4A in PDAC or normal pancreatic tissues detected by immunohistochemistry (IHC) (original magnification, 20×)(bar, 400 μm). **d** Specimens were scored and estimated in relative integrated optical density (IOD) value or in percentage of positive cells. **e** Western blotting of proteins extracted from eight paired samples of tumor (T) and normal pancreatic tissues (N). **f** Densitometric quantification of western blotting results. **g**, **h** UBL4A protein levels in HPDE and four PDAC cell lines detected by western blotting and densitometric quantification of western blotting results. **i** Relative. UBL4A mRNA levels in HPDE and four PDAC cell lines by qRT-PCR. The statistical significance between different groups was calculated with Student t-test. Data are shown as the mean ± SD of three replicates; *P < 0.05, **P < 0.01; ***P < 0.001; ns: not significant

**Incorrect** Fig. 7.

**Fig. 7:**
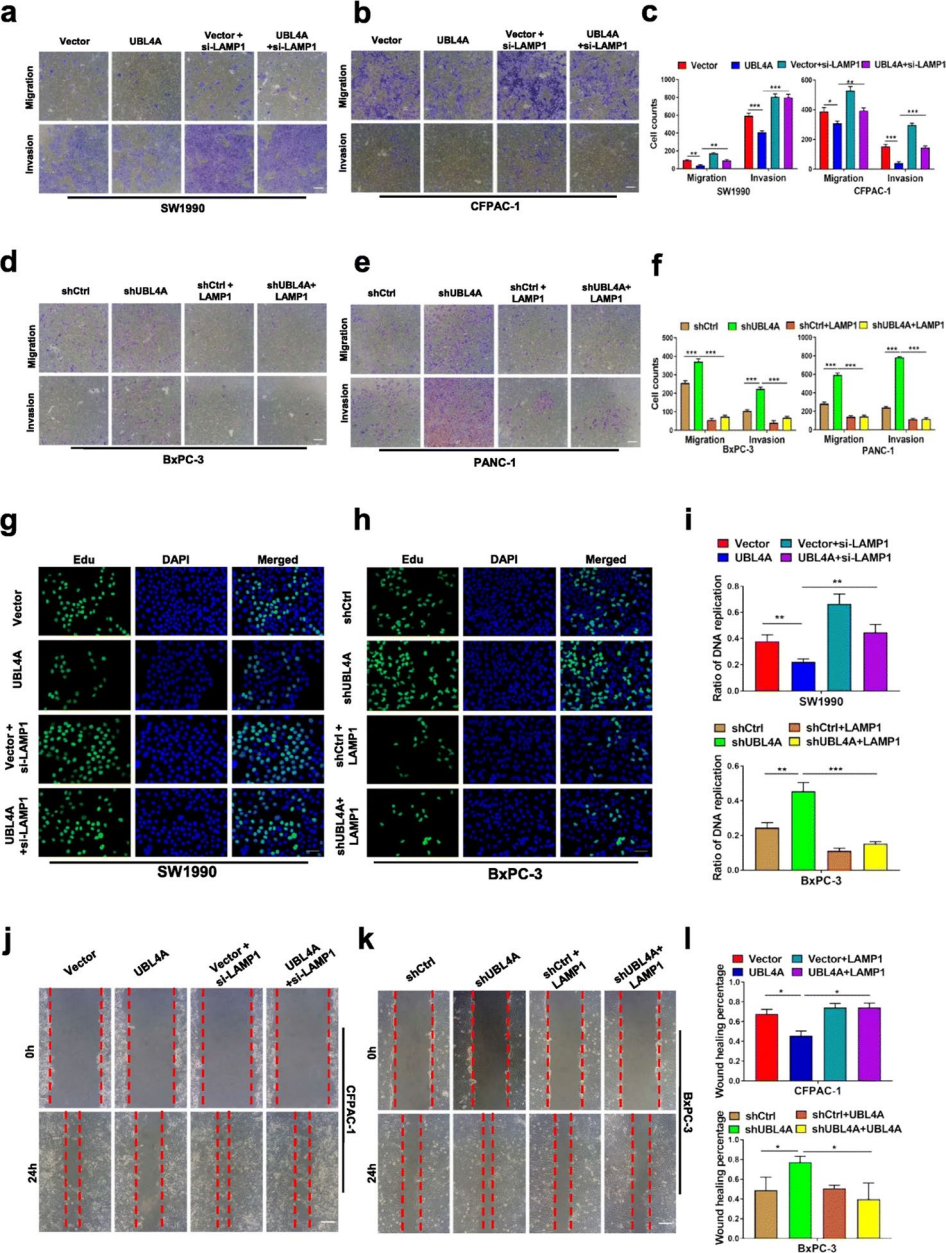
LAMP1 involves in UBL4A-mediated anti-tumor effects. **a**-**f** The role of LAMP1 in UBL4A-induced migration and invasion was demonstrated by transwell assay in four PDAC cell lines (original magnification, 10×) (bars, 25 μm). **g** The proliferative capacity of SW1990 was determined by EdU retention assays (original magnification, 20×) (bars, 50 μm) in four different groups (Vector, lv-UBL4A-Flag, siLAMP1 and lv-UBL4AFlag+siLAMP1). **h** Representative fluorescent photographs of Edu retention assay in BxPC-3 were subjected to shCtrl, LV-shUBL4A, LAMP1 plasmid and LV-shUBL4A + LAMP1 plasmid groups. **i** The ratio of DNA replication was calculated. **j**-**l** Wound healing assay was performed to detected the role of LAMP1 in UBL4A-mediated metastasis in CFPAC-1 and BxPC-3 (original magnification, 10×) (bars, 25 μm). The statistical significance between different groups was calculated with Student t- test. Data are shown as the mean ± SD of three replicates; *P < 0.05, **P < 0.01; ***P < 0.001; ns: not significant

**Correct** Fig. 7.

**Fig. 7:**
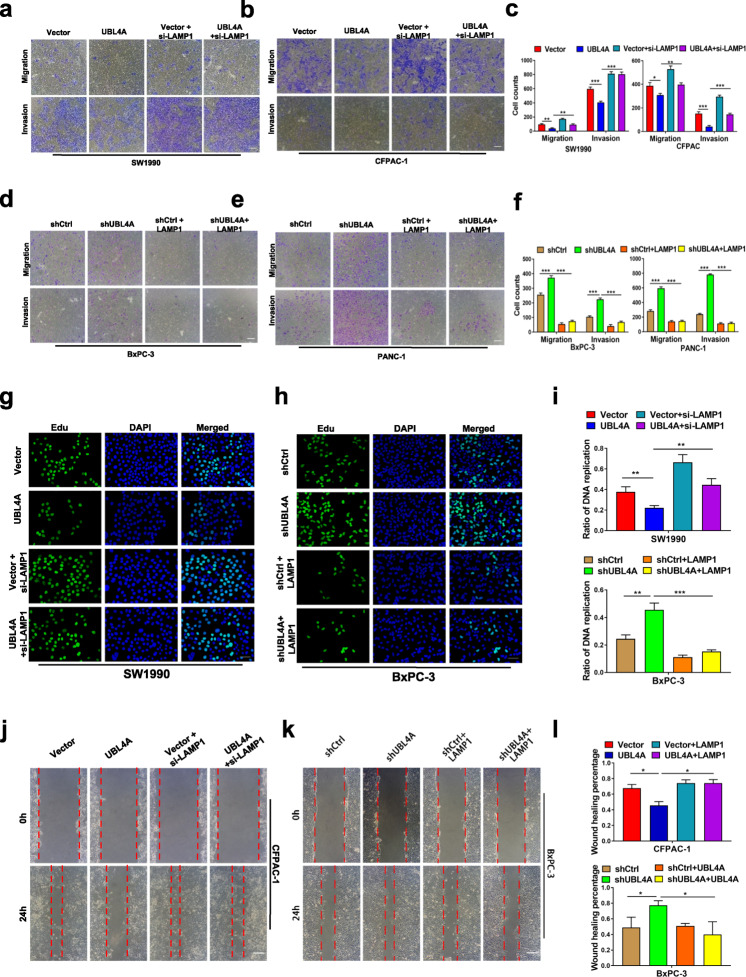
LAMP1 involves in UBL4A-mediated anti-tumor effects. **a**-**f** The role of LAMP1 in UBL4A-induced migration and invasion was demonstrated by transwell assay in four PDAC cell lines (original magnification, 10×) (bars, 25 μm). **g** The proliferative capacity of SW1990 was determined by EdU retention assays (original magnification, 20×) (bars, 50 μm) in four different groups (Vector, lv-UBL4A-Flag, siLAMP1 and lv-UBL4AFlag+siLAMP1). **h** Representative fluorescent photographs of Edu retention assay in BxPC-3 were subjected to shCtrl, LV-shUBL4A, LAMP1 plasmid and LV-shUBL4A + LAMP1 plasmid groups. **i** The ratio of DNA replication was calculated. **j**-**l** Wound healing assay was performed to detected the role of LAMP1 in UBL4A-mediated metastasis in CFPAC-1 and BxPC-3 (original magnification, 10×) (bars, 25 μm). The statistical significance between different groups was calculated with Student t- test. Data are shown as the mean ± SD of three replicates; *P < 0.05, **P < 0.01; ***P < 0.001; ns: not significant

**Incorrect** Fig. 8.

**Fig. 8:**
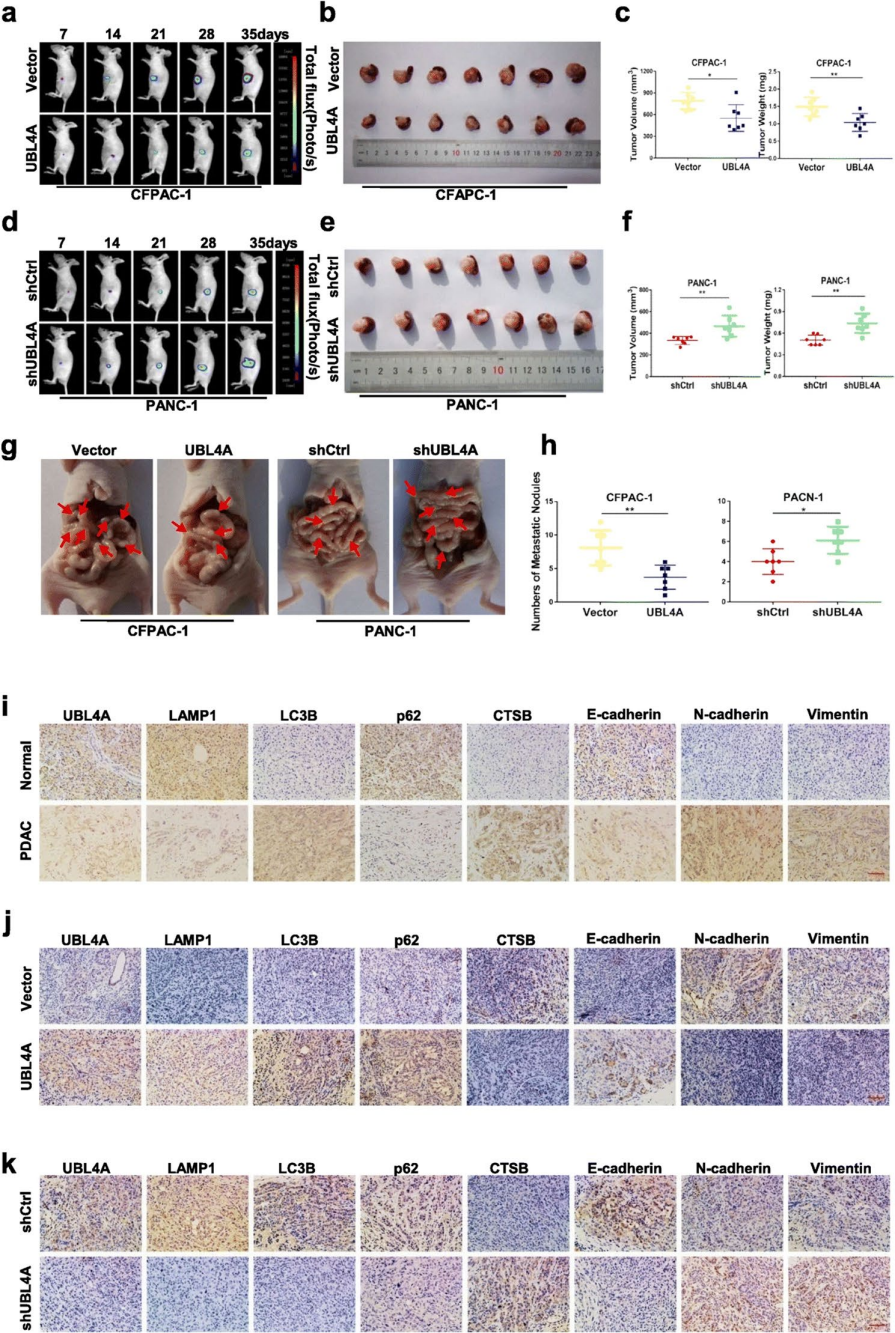
UBL4A inhibits tumor proliferation and metastasis in orthotopic tumor mode. **a**-**f** Representative bioluminescence imaging (following intraperitoneal injection of 0.1 mg/g luciferin) of mice at the day of 7, 14, 21, 28, and 35. At day 35, all mice were sacrificed and the primary tumors were removed, the tumor volume and weight was evaluated. **g**-**h** Representative images of orthotopic xenograft pancreatic cancer mouse models from four groups and metastatic nodes were calculated, red arrows indicated metastatic lesions. **i** The expression of UBL4A, LAMP1, LC3B, p62, CTSB, E-cadherin, N-cadherin and vimentin were analyzed by immunohistochemistry in human PDAC tissues and normal pancreas (original magnification, 20x) (bar, 400 μm) **j**-**k** The expression of UBL4A, LAMP1, LC3B, p62, CTSB, E-cadherin, N-cadherin and vimentin were analyzed in paraffin-embedded tissue sections of orthotopic pancreatic cancer models from different groups by immunohistochemistry (original magnification, 20x) (bars, 400 μm). The statistical significance between different groups was calculated with Student t-test. Data are shown as the mean ± SD of three replicates; *P < 0.05, **P < 0.01; ***P < 0.001; ns: not significant

**Correct** Fig. 8.

**Fig. 8:**
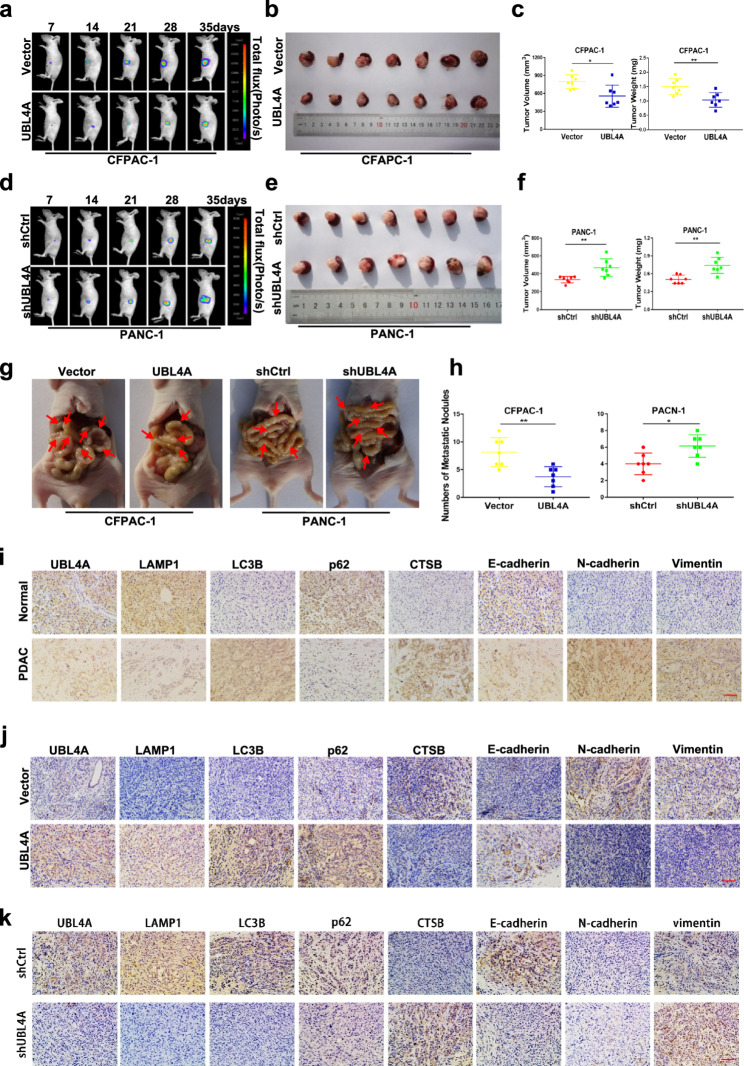
UBL4A inhibits tumor proliferation and metastasis in orthotopic tumor mode. **a**-**f** Representative bioluminescence imaging (following intraperitoneal injection of 0.1 mg/g luciferin) of mice at the day of 7, 14, 21, 28, and 35. At day 35, all mice were sacrificed and the primary tumors were removed, the tumor volume and weight was evaluated. **g**-**h** Representative images of orthotopic xenograft pancreatic cancer mouse models from four groups and metastatic nodes were calculated, red arrows indicated metastatic lesions. **i** The expression of UBL4A, LAMP1, LC3B, p62, CTSB, E-cadherin, N-cadherin and vimentin were analyzed by immunohistochemistry in human PDAC tissues and normal pancreas (original magnification, 20x) (bar, 400 μm) **j**-**k** The expression of UBL4A, LAMP1, LC3B, p62, CTSB, E-cadherin, N-cadherin and vimentin were analyzed in paraffin-embedded tissue sections of orthotopic pancreatic cancer models from different groups by immunohistochemistry (original magnification, 20x) (bars, 400 μm). The statistical significance between different groups was calculated with Student t-test. Data are shown as the mean ± SD of three replicates; *P < 0.05, **P < 0.01; ***P < 0.001; ns: not significant
