# Supplementary material for: UBL4A inhibits autophagy-mediated proliferation and metastasis of pancreatic ductal adenocarcinoma via targeting LAMP1
Source: J Exp Clin Cancer Res. 2019 Jul 9;38:297. doi: 10.1186/s13046-019-1278-9 (PMC6617940; doi:10.1186/s13046-019-1278-9)
Supplement: Supplementary file 1 — Table S1. The target sequences of lentiviruses and siRNAs used in transfection. (DOCX 14 kb) [file 13046_2019_1278_MOESM1_ESM.docx]

**Response Table 1: Original data of Fig. 1H**

| **Cell Lines** | **Experiment 1** | **Experiment 2** | | **Experiment 3** | ***P* value**  **(vs HPDE)** | |
| --- | --- | --- | --- | --- | --- | --- |
| HPDE | 0.815461 | 1.230527 | 1.193112 | | -- | |
| SW1990 | 0.277882 | 0.545395 | 0.511863 | | 0.0156 | |
| CFPAC-1 | 0.150985 | 0.275797 | 0.307772 | | 0.0041 | |
| PANC-1  BxPC-3 | 0.322559 | 0.585175 | | 0.556698 | | 0.0194 |
|  | 0.353575 | 0.699035 | | 0.647459 | | 0.0398 |
