# Supplementary material for: UBL4A inhibits autophagy-mediated proliferation and metastasis of pancreatic ductal adenocarcinoma via targeting LAMP1
Source: J Exp Clin Cancer Res. 2019 Jul 9;38:297. doi: 10.1186/s13046-019-1278-9 (PMC6617940; doi:10.1186/s13046-019-1278-9)
Supplement: Supplementary file 2 — Table S2. Sequences of primers used in Real-time RT-PCR. (DOCX 13 kb) [file 13046_2019_1278_MOESM2_ESM.docx]

**Response Table 2: Original data of Fig. 1I**

| **Cell Lines** | **Experiment 1** | **Experiment 2** | | **Experiment 3** | ***P* value**  **(vs HPDE)** | |
| --- | --- | --- | --- | --- | --- | --- |
| HPDE | 1.037284 | 1.120384 | 0.980372 | | -- | |
| SW1990 | 0.416797 | 0.387364 | 0.452837 | | 0.0002 | |
| CFPAC-1 | 0.371821 | 0.307264 | 0.402837 | | 0.0002 | |
| PANC-1  BxPC-3 | 0.630471 | 0.598273 | | 0.678273 | | 0.0013 |
|  | 0.827125 | 0.787362 | | 0.852937 | | 0.0115 |
