# Supplementary material for: UBL4A inhibits autophagy-mediated proliferation and metastasis of pancreatic ductal adenocarcinoma via targeting LAMP1
Source: J Exp Clin Cancer Res. 2019 Jul 9;38:297. doi: 10.1186/s13046-019-1278-9 (PMC6617940; doi:10.1186/s13046-019-1278-9)
Supplement: Supplementary file 3 — Table S3. Primary antibodies for WB, IHC, IF and co-IP. (DOC 38 kb) [file 13046_2019_1278_MOESM3_ESM.doc]

**Table S3. Primary antibodies for WB, IHC, IF and co-IP.**

| Antibody | Concentration  for WB | Concentration  for IHC | Concentration  for IF | Concentration  for co-IP | Specificity | Company |
| --- | --- | --- | --- | --- | --- | --- |
| UBL4A | 1:1000 | 1:100 | 1:50 | 5μg/ml | Rabbit polyclonal | Proteintech |
| E-cadherin | 1:1000 | 1:400 |  |  | Rabbit monoclonal | CST |
| N-cadherin | 1:500 | 1:200 |  |  | Mouse monoclonal | Santa Cruz |
| Vimentin | 1:5000 | 1:2000 |  |  | Mouse Monoclonal | Proteintech |
| LAMP1 | 1:1000 | 1:200 | 1:250 | 5μg/ml | Rabbit Polyclonal  Mouse Monoclonal  Rabbit Polyclonal | Proteintech Abcam  Abcam |
| LAMP2 | 1:1000 |  |  |  | Mouse Monoclonal | Proteintech |
| CTSB  LC3B | 1:1000  1:1000 | 1:200 | 1:200 |  | Rabbit Polyclonal  Rabbit Polyclonal  Rabbit Polyclonal | CST  Novus  CST |
|  |  | 1:100 |  |  | Rabbit Polyclonal | Proteintech |
| p62 | 1:1000 |  |  |  | Rabbit Polyclonal | CST |
|  |  | 1:100 |  |  | Rabbit Polyclonal | Proteintech |
