# Supplementary figures and images for: UBL4A inhibits autophagy-mediated proliferation and metastasis of pancreatic ductal adenocarcinoma via targeting LAMP1
Source: J Exp Clin Cancer Res. 2019 Jul 9;38:297. doi: 10.1186/s13046-019-1278-9 (PMC6617940; doi:10.1186/s13046-019-1278-9)

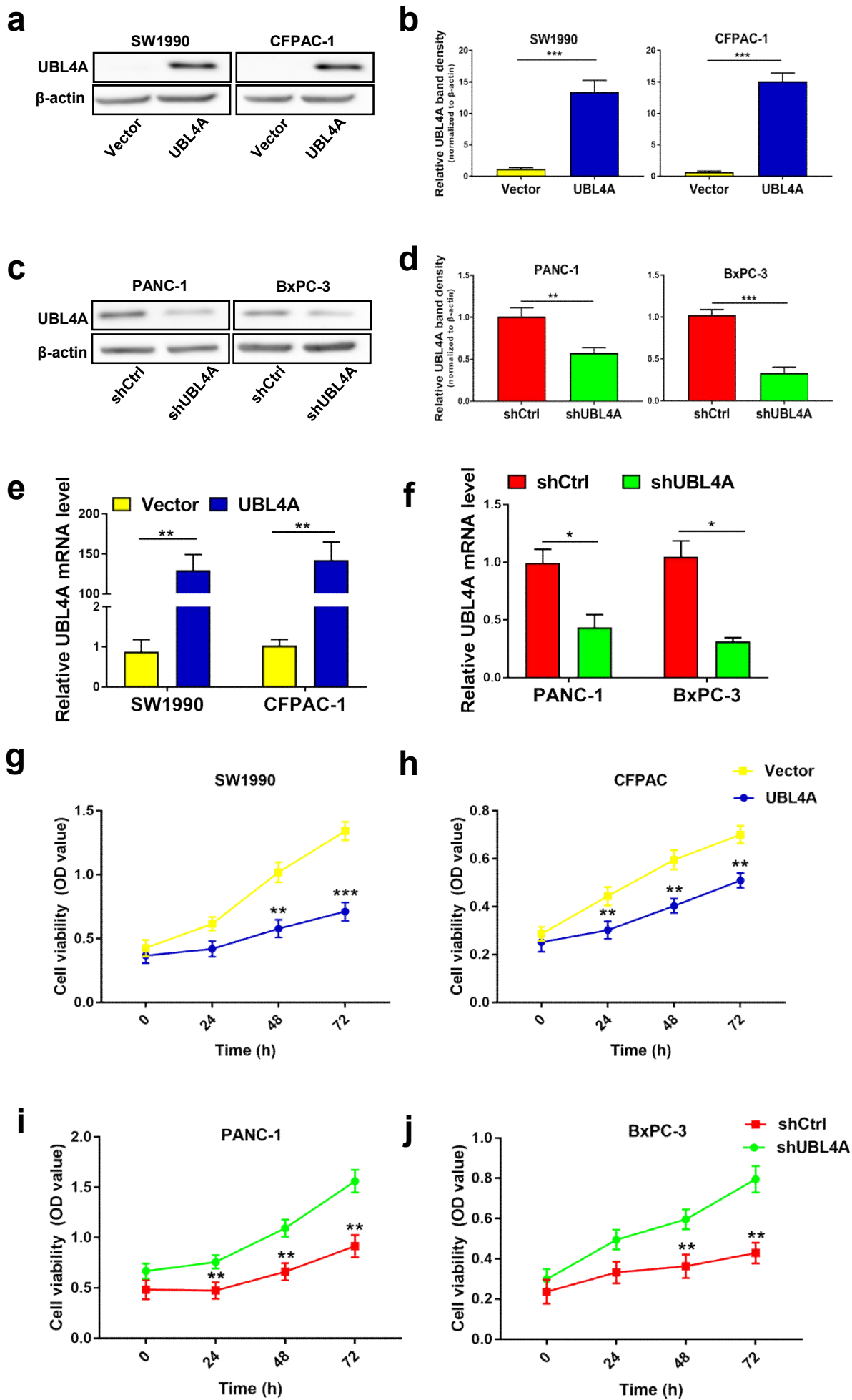

Supplement: Supplementary file 4 — Figure S1. The efficiency of UBL4A downregulation and upregulation is analyzed and UBL4A inhibits tumor proliferation in PDAC. a, b The efficiency of UBL4A overexpression (LV-UBL4A-flag) in SW1990 and CFPAC-1 was detected by western blotting. c, d The knockdown efficiency of LV-shUBL4A in PANC-1 and BxPC-3 was analyzed by western blotting. e, f The efficiency of UBL4A downregulation and upregulation was analyzed by qRT-PCR in four PDAC cell lines. g-j Proliferation rate was analyzed by CCK-8 assay of indicated SW1990, CFPAC-1 (Vector, LV-UBL4A) and PANC-1, BxPC-3 (shCtrl, LV-shUBL4A). The statistical significance between different groups was calculated with Student t-test. Data are shown as the mean ± SD of three replicates; *P < 0.05, **P < 0.01; ***P < 0.001; ns: not significant. (PDF 291 kb) [file 13046_2019_1278_MOESM4_ESM.pdf]

**a**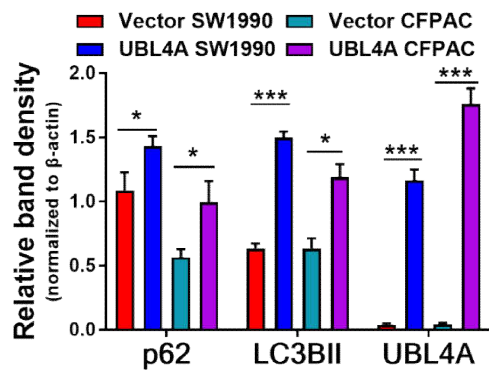**b**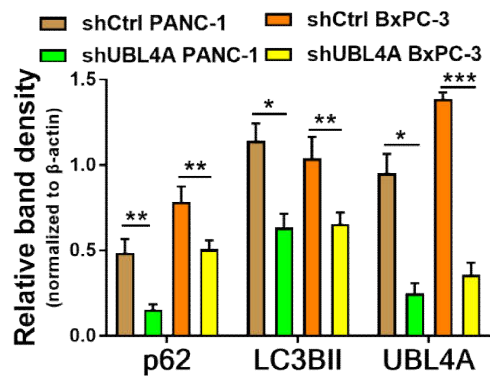**c**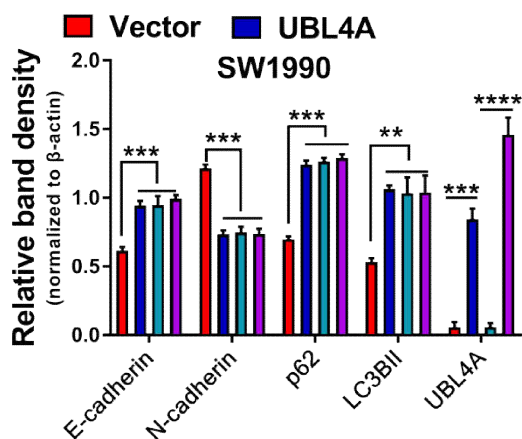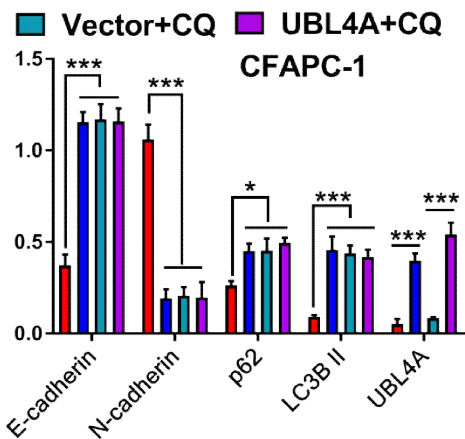**d**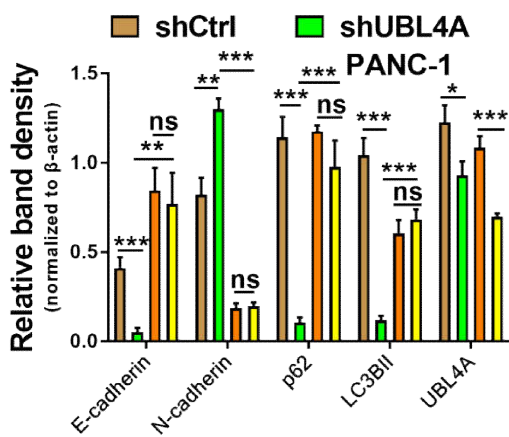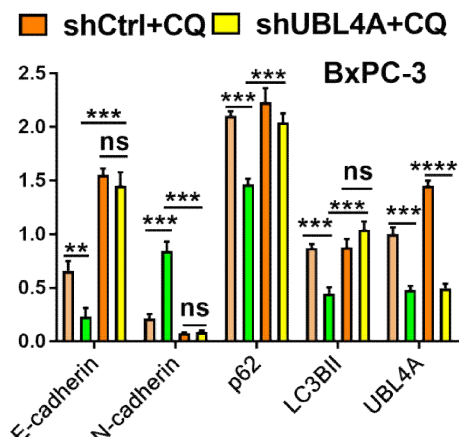

Supplement: Supplementary file 5 — Figure S2. The relative band density of results in western blotting. a, b The expression of LC3B and p62 in four PDAC cell lines of different groups (Vector, LV-UBL4A-Flag, shCtrl, LV-shUBL4A) were calculated. c The relative band density of E-cadherin, N-cadherin, p62, LC3B and UBL4A in SW1990 and CFPAC in four different groups. d The relative band density of E-cadherin, N-cadherin, p62, LC3B and UBL4A in PANC-1 and BxPC-3 in four different groups. The statistical significance between different groups was calculated with Student t-test. Data are shown as the mean ± SD of three replicates; *P < 0.05, **P < 0.01; ***P < 0.001; ns: not significant. (PDF 334 kb) [file 13046_2019_1278_MOESM5_ESM.pdf]

**a**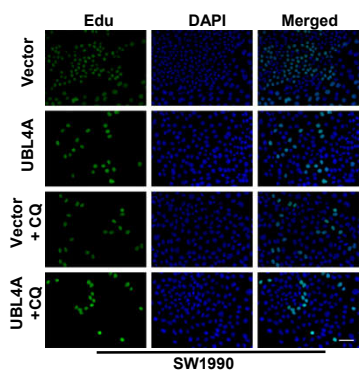**b**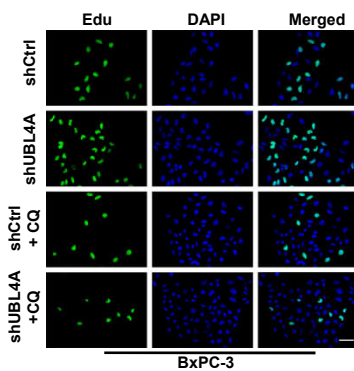**c**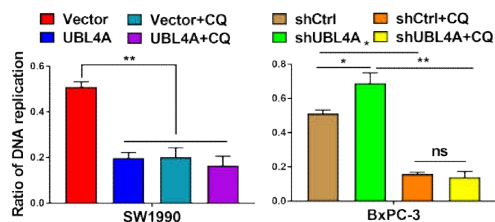**d**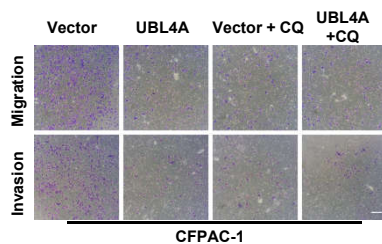**e**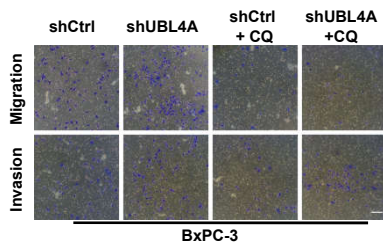**f**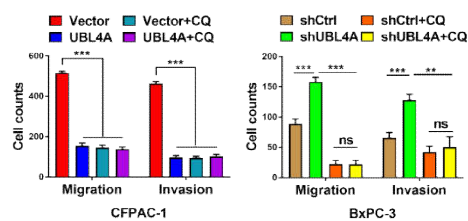**g**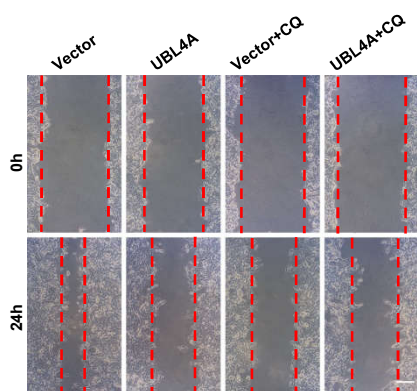**h**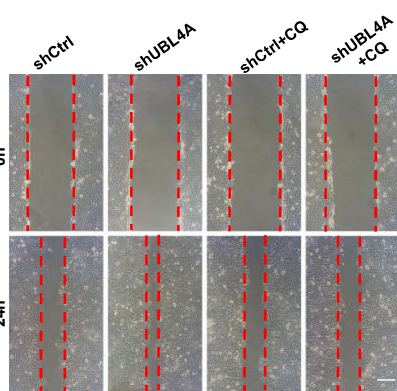**i**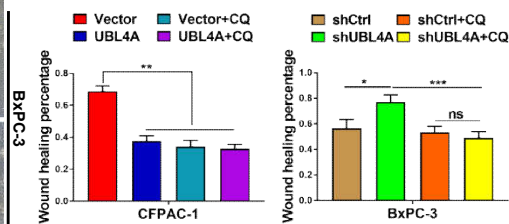

Supplement: Supplementary file 6 — Figure S3. UBL4A-induced inhibition of tumor depends on autophagy. a-c The proliferative capacity of pancreatic cancer cells was determined by EdU retention assays (original magnification, 20×) (bars, 50 μm) and the ratio of DNA replication was calculated. d-f The role of CQ in UBL4A-induced migration and invasion was demonstrated by transwell assay in CFPAC-1 and BxPC-3 (original magnification, 10×) (bars, 25 μm). g-i Wound healing assay was performed to detected the role of CQ in UBL4A-mediated metastasis in CFPAC-1 and BxPC-3 (original magnification, 10×) (bars, 25 μm). The statistical significance between different groups was calculated with Student t-test. Data are shown as the mean ± SD of three replicates; *P < 0.05, **P < 0.01; ***P < 0.001; ns: not significant. (PDF 259 kb) [file 13046_2019_1278_MOESM6_ESM.pdf]

**a**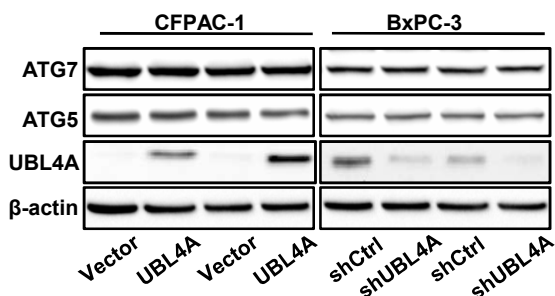**b**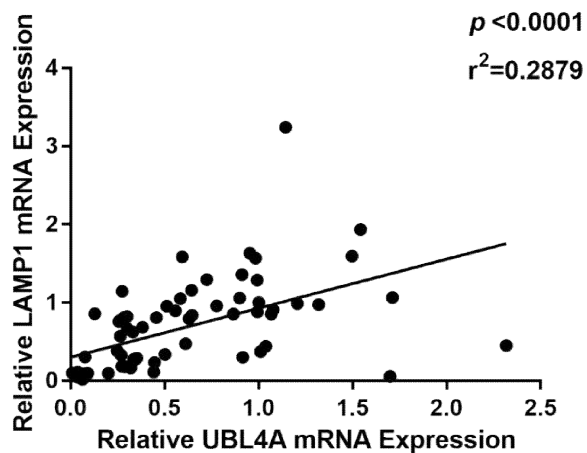**c**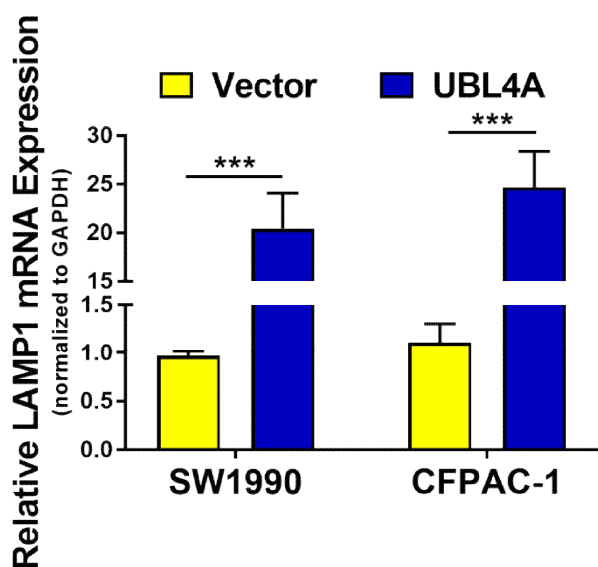**d**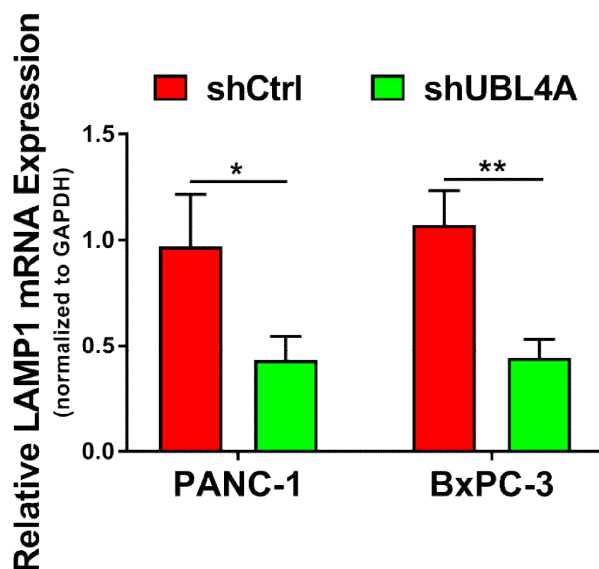**e**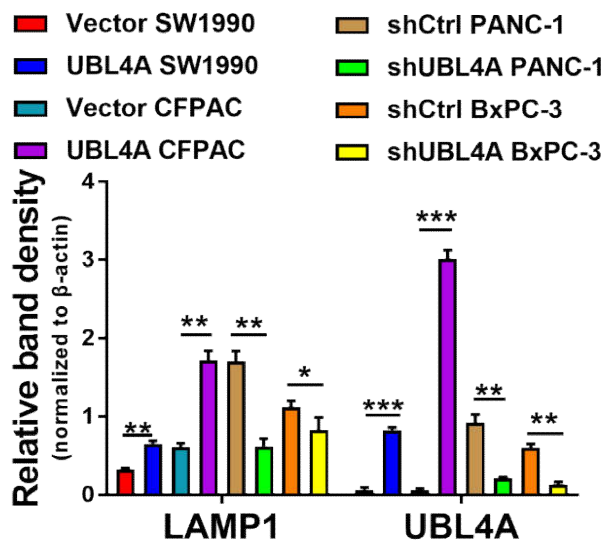

Supplement: Supplementary file 7 — Figure S4. UBL4A-induced Autophagy inhibition is related with LAMP1 rather than ATG5 or ATG7. a The expression of ATG5 and ATG7 in CFPAC-1 and BxPC-3 of different groups (Vector, LV-UBL4A-Flag, shCtrl, LV-shUBL4A) were analyzed by western blotting. b qRT-PCR assays examined the expression of both UBL4A and LC3B mRNA in each of 69 PDAC tissues, and the relevance was listed in each blot (r2 = 0.2879, P < 0.0001). c-d qRT-PCR analyses of LAMP1 in four PDAC cell lines of different groups (Vector, LV-UBL4A-Flag, shCtrl, LV-shUBL4A). e The expressions of LAMP1 and UBL4A in four PDAC cell lines of different groups (Vector, LV-UBL4A-Flag, shCtrl, LV-shUBL4A) were calculated. The statistical significance between different groups was calculated with Student t-test. Data are shown as the mean ± SD of three replicates; *P < 0.05, **P < 0.01; ***P < 0.001; ns: not significant. (PDF 279 kb) [file 13046_2019_1278_MOESM7_ESM.pdf]

**a**

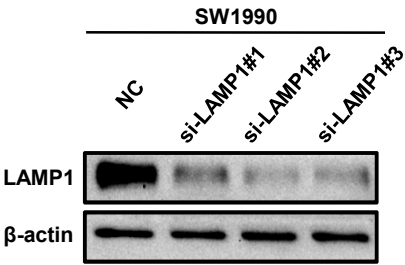

**b**

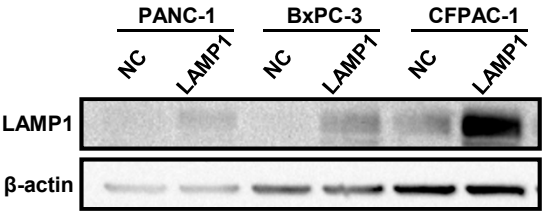

**c**

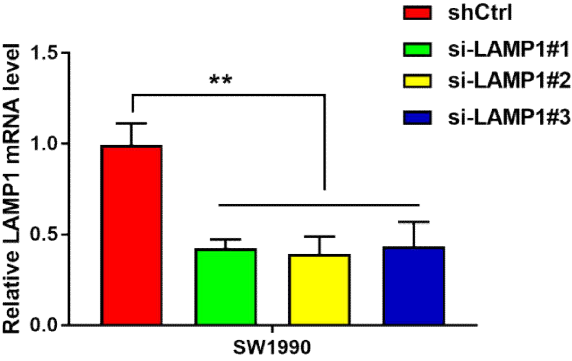

**d**

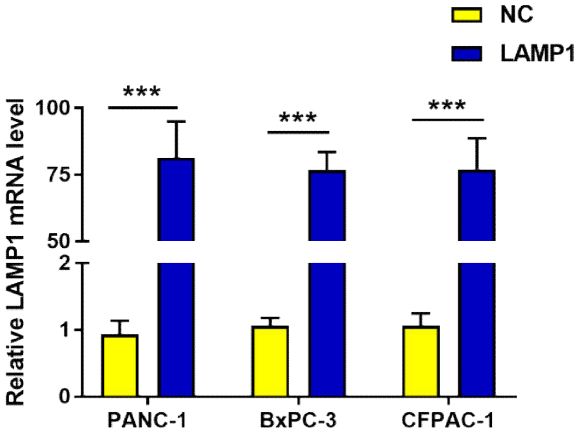

**e**

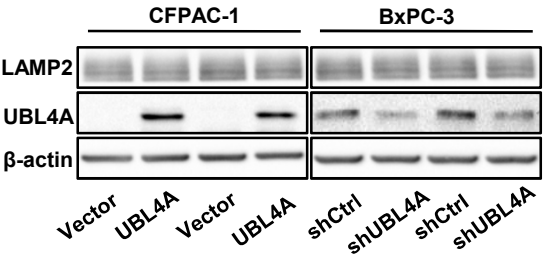

Supplement: Supplementary file 8 — Figure S5. The efficiency of LAMP1 downregulation and upregulation is detected by western blot and qRT-PCR. a-b The efficiency of LAMP1 downregulation and upregulation was detected by western blotting. c-d The efficiency of LAMP1 downregulation and upregulation was detected by qRT-PCR. e The expression of LAMP2 in CFPAC-1 and BxPC-3 of different groups (Vector, LV-UBL4A-Flag, shCtrl, LV-shUBL4A) were analyzed by western blotting. The statistical significance between different groups was calculated with Student t-test. Data are shown as the mean ± SD of three replicates; *P < 0.05, **P < 0.01; ***P < 0.001; ns: not significant. (PDF 210 kb) [file 13046_2019_1278_MOESM8_ESM.pdf]
