# Supplementary material for: UBL4A inhibits autophagy-mediated proliferation and metastasis of pancreatic ductal adenocarcinoma via targeting LAMP1
Source: J Exp Clin Cancer Res. 2019 Jul 9;38:297. doi: 10.1186/s13046-019-1278-9 (PMC6617940; doi:10.1186/s13046-019-1278-9)
Supplement: Supplementary file 9 — Supplementary Methods. (DOCX 25 kb) [file 13046_2019_1278_MOESM9_ESM.docx]

**Supplementary Methods**

**Cell lines and reagents**

The human pancreatic cancer cell lines BxPC-3 and PANC-1 and human pancreatic duct epithelial cells (HPDE) were purchased from the American type culture collection. CFPAC-1 and SW1990 were bought from the Type Culture Collection of the Chinese Academy of Sciences, Shanghai Institute of Cell Biology, Chinese Academy of Sciences (Shanghai, China). BxPC-3 and PANC-1 cell lines were routinely cultured in RPMI 1640 medium (GE Healthcare Life Sciences HyClone Laboratoris, Logan, Utah, USA), and CFPAC-1, SW1990 and HPDE cell lines were grown in Dulbecco’s modified Eagle’s medium (Gibco, Thermo Fisher Scientific, Grand Island, NY, USA) supplemented with 10% FBS (BIONTR, Cordoba, ARG), penicillin (100 U/ml) and streptomycin (100 mg/ml). All cells were cultured at 37 °C with 5% CO2.

**Wound healing assay**

The wound healing assay was performed as described previously [1, 2]. Briefly, transfected cells were set up at 2 × 10^5^ cells per well in a 6-well plate and allowed to grow to converge. Cells were washed three times in medium and pretreated with mitomycin C (10 μg/mL) 2 h before an artificial “wound” created with a 200μL pipettetip at 0h and then incubated in 1% serum medium. Images were acquired at 0h and 24h at 20× on Olympus microscope. The percentage of wound closure was estimated by Image J software.

**Migration and invasion assays**

Migration and invasion assays were performed as described previously [1, 2]. The motility and invasiveness of cells exposed to DAC were assayed using 8 μm pore size Falcon® inserts precoated or uncoated with Matrigel (BD Biosciences, San Jose, CA, USA). Culture medium containing 10% FBS was added to lower chambers and 5-10 × 10^4^ cells in 200μl of FBS-free medium were seeded into upper chambers. After incubation for 24 h at 37 °C, non-migrated or non-invaded cells were removed from the upper surface of the filter with a cotton swab. Invasive cells on the bottom surface of the membrane were fixed in methanol and then stained with crystal violet. The number of cells in five randomly selected fields (20×) was counted, and each experiment was repeated at least three times.

**Colony formation assay**

The colony formation assay was performed as described previously [1, 2]. Five-hundred cells transfected with Lv-shUBL4A and Lv-shCtrl as well as Lv-UBL4A-Flag and lentiviral empty vector were cultured in 6-well plates. The medium was changed every 3 days. After day 14, the colonies were counted after fixation in methanol for 10 min with 1% crystal violet staining. The colonies were counted manually in five fields (10×, Olympus).

***In vitro* proliferation**

Cell proliferation was examined using Cell Counting Kit-8 (CCK-8, Dojindo Laboratories, Kumamoto, Japan) according to the manufacturer’s instructions. Cells (5×10^3^ cells per well) were seeded into a 96-well plate. Absorbance was measured daily for 3 consecutive days at 450 nm (ELx808, BioTek, USA).

**EdU proliferation assay**

Cell proliferation was also determined by a 5-ethynyl-2’-deoxyuridine (EdU) assay (RIBOBIO, Guangzhou, China).The EdU proliferation assay was performed as described previously [2]. Following transfection, 1 × 10^4^ infected cells of each group were seeded in 6-well plates, and then, the cells were treated following the manufacturer’s instructions. The cells were visualized with a fluorescence microscope (20×, Olympus).

**Western blotting**

The methodology has been described previously [2, 3]. In brief, total proteins from pancreatic cancer cells or tissues were extracted using RIPA buffer (Beyotime Institute of Biotechnology, Beijing, China) that contained protease inhibitor cocktail and phosphatase inhibitor and homogenized pancreatic tissues or cells were homogenized in protein lysate buffer that contained protease inhibitor and phosphatase inhibitor (Roche, Shanghai, China) and debris was removed by centrifugation. The samples were resolved on SDS-PAGE and electrophoretically transferred onto polyvinylidene difluoride (PVDF) (Invitrogen, Carlsbad, CA, USA). The membranes were blocked with 5% skimmed milk, incubated with the appropriate primary antibodies (Table S3) and horseradish peroxidase-conjugated secondary antibodies (1:2000; ZSGB-BIO). The bands were visualized with the Molecular Imager System (BIO-RAD, Hercules, USA) using an enhanced chemiluminescence method (Thermo Fisher Scientific). β-actin was used as the protein loading control and the level of protein expression was calibrated as the relative band density to that of β-actin.

**Immunohistochemical staining**

The immunohistochemical staining protocol has been described previously [4, 5]. The paraffin-embedded tissue sections (5 mm) were immunostained with the appropriate primary antibodies (Table S3). The numbers of positive cells were counted in five high-power fields using a microscope (20×, Olympus).

**References:**

1. Hu J, Li L, Chen H, Zhang G, Liu H, Kong R et al. MiR-361-3p regulates ERK1/2-induced EMT via DUSP2 mRNA degradation in pancreatic ductal adenocarcinoma. Cell Death Dis 2018; 9: 807.

2. Li L, Chen H, Gao Y, Wang YW, Zhang GQ, Pan SH et al. Long Noncoding RNA MALAT1 Promotes Aggressive Pancreatic Cancer Proliferation and Metastasis via the Stimulation of Autophagy. Molecular Cancer Therapeutics 2016; 15: 2232-43.

3. Ji L, Li L, Qu F, Zhang G, Wang Y, Bai X et al. Hydrogen sulphide exacerbates acute pancreatitis by over-activating autophagyvia AMPK/mTOR pathway. Journal of Cellular and Molecular Medicine 2016; 20: 2349-61.

4. Lv JC, Wang G, Pan SH, Bai XW, Sun B. Lycopene protects pancreatic acinar cells against severe acute pancreatitis by abating the oxidative stress through JNK pathway. Free Radic Res 2015; 49: 151-63.

5. Wang Y, Zhou Y, Jia G, Han B, Liu J, Teng Y et al. Shikonin suppresses tumor growth and synergizes with gemcitabine in a pancreatic cancer xenograft model: Involvement of NF-kappaB signaling pathway. Biochem Pharmacol 2014; 88: 322-33.
